# Adolescent and Parent Perspectives on Digital Phenotyping in Youths With Chronic Pain: Cross-Sectional Mixed Methods Survey Study

**DOI:** 10.2196/47781

**Published:** 2024-01-11

**Authors:** Bridget A Nestor, Justin Chimoff, Camila Koike, Elissa R Weitzman, Bobbie L Riley, Kristen Uhl, Joe Kossowsky

**Affiliations:** 1 Department of Anesthesiology, Critical Care, and Pain Medicine Boston Children’s Hospital Boston, MA United States; 2 Department of Anesthesia Harvard Medical School Boston, MA United States; 3 Division of Adolescent and Young Adult Medicine Boston Children's Hospital Boston, MA United States; 4 Department of Pediatrics Harvard Medical School Boston, MA United States; 5 Division of Addiction Medicine Boston Children's Hospital Boston, MA United States; 6 Department of Psychosocial Oncology and Palliative Care Dana Farber Cancer Institute Boston, MA United States; 7 Department of Psychiatry Boston Children’s Hospital Boston, MA United States; 8 Division of Sleep Medicine Harvard Medical School Boston, MA United States

**Keywords:** acceptability, adolescent, chronic pain, digital phenotyping, mobile health, pediatric

## Abstract

**Background:**

Digital phenotyping is a promising methodology for capturing moment-to-moment data that can inform individually adapted and timely interventions for youths with chronic pain.

**Objective:**

This study aimed to investigate adolescent and parent endorsement, perceived utility, and concerns related to passive data stream collection through smartphones for digital phenotyping for clinical and research purposes in youths with chronic pain.

**Methods:**

Through multiple-choice and open-response survey questions, we assessed the perspectives of patient-parent dyads (103 adolescents receiving treatment for chronic pain at a pediatric hospital with an average age of 15.6, SD 1.6 years, and 99 parents with an average age of 47.8, SD 6.3 years) on passive data collection from the following 9 smartphone-embedded passive data streams: accelerometer, apps, Bluetooth, SMS text message and call logs, keyboard, microphone, light, screen, and GPS.

**Results:**

Quantitative and qualitative analyses indicated that adolescents and parent endorsement and perceived utility of digital phenotyping varied by stream, though participants generally endorsed the use of data collected by passive stream (35%-75.7% adolescent endorsement for clinical use and 37.9%-74.8% for research purposes; 53.5%-81.8% parent endorsement for clinical and 52.5%-82.8% for research purposes) if a certain level of utility could be provided. For adolescents and parents, adjusted logistic regression results indicated that the perceived utility of each stream significantly predicted the likelihood of endorsement of its use in both clinical practice and research (*P*_s_<.05). Adolescents and parents alike identified accelerometer, light, screen, and GPS as the passive data streams with the highest utility (36.9%-47.5% identifying streams as useful). Similarly, adolescents and parents alike identified apps, Bluetooth, SMS text message and call logs, keyboard, and microphone as the passive data streams with the least utility (18.5%-34.3% identifying streams as useful). All participants reported primary concerns related to privacy, accuracy, and validity of the collected data. Passive data streams with the greatest number of total concerns were apps, Bluetooth, call and SMS text message logs, keyboard, and microphone.

**Conclusions:**

Findings support the tailored use of digital phenotyping for this population and can help refine this methodology toward an acceptable, feasible, and ethical implementation of real-time symptom monitoring for assessment and intervention in youths with chronic pain.

## Introduction

Digital phenotyping (or personal sensing [1]) refers to the continuous, passive collection of social and behavioral data, often gathered through smartphone sensors, allowing for ecologically valid insight into an individual’s daily life [2]. In recent years, this method of assessment has been implemented in various populations to measure patterns of mental health symptoms, sleep, activity, and other social behaviors [3,4]. Although this methodology holds great promise for capturing moment-to-moment data that can inform individually adapted and timely interventions, whether individuals feel comfortable with this style of data collection remains largely undetermined. This study investigates adolescent and parental attitudes toward passive data streams collected through smartphone for digital phenotyping in a sample of treatment-seeking youths with chronic pain.

For youths with chronic pain, digital phenotyping represents a promising means of both assessment and intervention, as smartphone ownership or access has become nearly universal in adolescence [5]. Detailed, in-clinic assessments of youths with chronic pain may ask adolescents and their parents to report on changes in their pain, activity, sleep, substance use, and other psychological symptoms to capture a biopsychosocial picture of the patient over time. Other types of assessments may use, exclusively or partially, survey data collected from participants on their smartphones. Whether through surveys or in-person, providing accurate reports of many of these features relevant to chronic pain is quite difficult, and studies have shown that retrospective bias obscures reports of mood [6], sleep [7], and activity [8]. As such, digital phenotyping may offer more accurate, sensitive, and objective symptom information for assessment. Moreover, youths with chronic pain may also be well-suited for digital, just-in-time adaptive interventions [9], as these interventions can be tailored for patients based on the continuous monitoring of the complex longitudinal interplay of their physical and mental health symptoms.

Indeed, passive data streams embedded in smartphones, including accelerometers, GPS sensors, call logs, and light sensors, among others, can continuously collect various metrics of an individual’s in situ data, holding massive information about social and behavioral patterns. Previous studies have demonstrated that data collected through these streams correlate with validated self-report measures of physical and mental health across different populations, including college students with anxiety, patients undergoing chemotherapy, adults with schizophrenia, and persons with HIV [3,10-16]. More recently, this approach has been extended to youths, indicating that passive data streams collected through smartphone are significantly associated with internalizing symptoms in children and adolescents [17]. Passive data collection can also be combined with more active prompts to promote health behaviors, such as coping or mindfulness.

Findings from previous research highlight the ecological validity of digital phenotyping methodology and its capacity to provide true “snapshots” of an individual’s real life. This approach does not rely on any participant report, which is often subject to recall bias [18], and for adolescents in particular, digital phenotyping assessment also alleviates the burden of parental report of adolescent symptom changes, reducing informant effects [19]. In addition, digital phenotyping allows for data collection in a person’s own environment, thus diminishing issues related to the limited generalizability of laboratory-based findings [20]. Finally, digital phenotyping is unobtrusive; beyond the initial downloading of an app for passive data collection, no participant action is required, distinguishing it from other technologically advanced approaches to data collection, such as ecological momentary assessment, which holds high ecological validity but requires ongoing and somewhat burdensome participant engagement [21].

Despite the methodological strengths and appeal of digital phenotyping for assessment and intervention, concerns exist about its implementation for clinical and research purposes. At the forefront of these concerns are hesitations about patient privacy, confidentiality, and data security. Are individuals willing to consent to passive data streams on their smartphone collecting extensive personal data? Would individuals endorse the collection of some types of personal data but not others? Research into the general acceptability of digital phenotyping lags behind its implementation, though studies have begun to investigate this topic in more depth, revealing varying levels of acceptability as well as concerns, though findings describe adults [22,23]. Investigation of acceptability is also necessary for pediatric populations, for whom parental consent and youths’ assent to data collection are required.

To that end, this study investigates adolescent and parent perspectives on endorsement, perceived utility, and concerns related to passive data stream collection through smartphone for digital phenotyping for clinical and research purposes in youths with chronic pain. In particular, we assessed participant perspectives on passive data collection through smartphone from the following 9 streams: screen, light, apps and installations, GPS, accelerometer, SMS text message and call logs, keyboard, Bluetooth, and microphone. We hypothesized that the majority of adolescents and their parents would endorse the use of digital phenotyping and that they would identify this methodology as useful. We further anticipated that participants’ perceived utility of each passive data stream would predict their endorsement of the stream’s use, and that this would be true for both adolescents and parents in the adolescent-parent dyads. Finally, we expected that adolescents and parents would express concerns about privacy and data security.

## Methods

This study was cross-sectional and adopted a mixed methods approach.

### Recruitment and Procedure

This study was conducted for 15 months, from June 2021 to September 2022. Participants were recruited for study participation primarily at clinic visits and secondarily through other outreach (eg, letter, email, or secure patient portal message). Specifically, a member of the study team reviewed weekly pain clinic schedules at the hospital to identify eligible families with upcoming scheduled appointments (either in-person or through Zoom [Zoom Video Communications] telehealth) for study recruitment. The study team then contacted the clinical care team to obtain approval to briefly join potentially eligible patient appointments. With approval from clinical care providers, the study team then met with eligible families after the clinical portion of their appointment (either in-person or through Zoom) to share information about the study, answer any questions, and obtain verbal consent from a parent and assent from the adolescent patient. In addition, some eligible families were contacted through letter, email, or secure patient portal message to participate in the study and also given the option to opt out of participation and additional contact. If families did not opt out within 3 days, a member of the study team contacted them by phone to further explain the study, answer relevant questions, and obtain verbal informed consent. A total of 2 voicemails, with a standard script of study description, were left for families if they could not be contacted directly. If no response was received after 2 days, families were considered not interested in participating and were not contacted again.

### Participants

Participants (ie, adolescent patients and their parents) were recruited from a pain treatment clinic at a pediatric hospital in the Northeast United States. Adolescent patients (n=103) were included in the study if they were (1) between 13 and 18 years of age and (2) receiving treatment for chronic pain at the pediatric hospital. Parents (n=99) were included if their child was eligible for study participation. All participants were able to understand and respond to questions in English. Of those approached for participation, 10 refused to participate due to disinterest in research or concerns about the time commitment of study participation. One participant dropped out after consenting to the study due to concerns about the time commitment of completing the survey.

### Ethical Considerations

Institutional review board (IRB) approval (protocol number IRB-P00035845) was obtained in November 2020 to access protected health information in order to determine participant eligibility and conduct this study. Boston Children’s Hospital (BCH) gave ethical approval for the study. The senior author (JK) served as the responsible principal investigator for this study. All patients provided informed consent (assent from adolescents and consent from parents). Of note, only verbal consent was obtained for this study, not written consent, because the BCH IRB determined this study met the exemption for written consent. This decision was determined because this study was deemed to be of no more than minimal risk of harm to participants and involved no procedures for which written consent is normally required outside of the research context. A member of the study team sent a secure survey link to each participating adolescent and parent who provided their respective verbal assent and consent by phone. Participants completed their surveys through Research Electronic Data Capture (REDCap; Vanderbilt University) [24,25], a secure, Health Insurance Portability and Accountability Act (HIPAA)–compliant, web-based application that collects and stores data. The data were deidentified. Participants were compensated with US $15 Amazon gift cards for their study participation.

### Measures

Each participant completed a REDCap survey comprising demographic questions and then watched 9 short videos created by the study team, which described what each data stream does, explained what information would be collected from each stream, displayed what collected information would look like, and gave a couple of examples of how each data stream may be useful for participants’ health care, specifically their pain assessment and treatment. Participants then answered multiple-choice and free-response questions about each passive data stream following each video. Demographic questions assessed who was completing the survey (eg, mother, father, guardian, or patient), their age, education, type of phone used, and comfort with using technology. Adolescent and parent comfort with technology was assessed on a 5-point Likert scale (eg, 1=very uncomfortable; 2=uncomfortable; 3=somewhat comfortable; 4=comfortable; and 5=very comfortable).

Between 4 and 9 multiple-choice questions probed adolescent and parent endorsement, perceived utility, and concerns related to each passive data stream. Participants also had the option to answer free-response questions about each stream. Specifically, the survey investigated adolescents’ and parents’ attitudes toward 9 passive data streams: accelerometer, apps, SMS text message and call logs, Bluetooth, keyboard, microphone, light, screen, and GPS. The following main outcomes were assessed: (1) endorsement was measured as participants’ “yes” versus “no” response to having data from a particular passive data stream shared with their doctor for treatment planning, for research purposes, or with their parents; (2) perceived utility was measured on a 5-point Likert scale that queried how useful the information from a particular passive data stream would be in helping to understand how patients are doing (eg, 1=not useful at all; 2=a little useful; 3=somewhat useful; 4=useful; and 5=very useful); (3) perceived concerns were measured on a 5-point Likert scale to determine how strongly participants agreed with a given concern for a specific passive data stream (eg, 1=strongly disagree; 2=disagree; 3=neutral; 4=agree; and 5=strongly disagree)*.* In addition, adolescents were also asked whether or not they would endorse their passively collected data being shared with their parents. Concerns probed for each passive data stream varied, ranging from items querying the perceived accuracy and validity of the data collected to items assessing participant comfort with privacy or data security. Participants were also given the option to provide open-ended responses regarding any additional stream-specific concerns. Of note, all items were presented to both adolescents and parents, with slight word changes depending on whether items were adolescent-facing or parent-facing (eg, “I don’t think this passive data stream would be useful for my treatment” vs “I don’t think this passive data stream would be useful for my child’s treatment”). In this article, we present item wording only for adolescent-facing questions. See Multimedia Appendix 1 for all survey items, which were created by the study team. The survey took approximately 30 minutes for participants to complete.

### Data Analysis

This study was a content analysis with both quantitative and qualitative aspects.

#### Quantitative Analysis

SPSS software (version 28; IBM Corp) was used to conduct descriptive and inferential data analyses of endorsement, perceived utility, and concerns. We conducted separate binary logistic regression analyses for adolescents and for parents in order to statistically test whether perceived utility predicted endorsement of passive data streams for clinical practice and research. In addition, to account for interdependence within each adolescent and parent dyad, we also conducted dyadic analyses. We assessed for dyadic interdependence in primary outcome variables (ie, adolescent and parent endorsement for each passive data stream for clinical practice and research) with pairwise intraclass correlation coefficients [26]. For outcome variables that suggested significant dyadic interdependence, we conducted multilevel binary logistic regression analyses. Specifically, at the level of the dyad, we tested whether the perceived utility of each passive data stream predicted endorsement for clinical and research use. We also tested for within-dyad differences predicting endorsement and for within-dyad differences for perceived utility predicting endorsement.

#### Qualitative Analysis

An inductive coding process [27], which is considered an efficient methodology for qualitative data, was used to conduct qualitative analysis of open-ended responses in Microsoft Excel (Microsoft Corp). Our multistage approach involved 2 members of the study team independently reviewing open-ended responses, creating a coding framework, and coding responses into themes for thematic saturation. Coding frameworks and coded responses were then reviewed for consensus standard between study members. Discrepancies in coding responses for themes were rare (n=6) and resolved by discussion with an additional study team member. Each element of a given response was coded separately by theme. For example, if 1 response contained a concern about privacy and a concern about data, this response was considered to have 2 separate concerns. In addition, concerns were counted separately. For example, if a participant reported 2 distinct concerns about GPS, each concern counted separately. See Table S1 in Multimedia Appendix 2 for the codebook used in this study.

## Results

### Sample Characteristics

The sample consisted of 103 adolescents (mean age 15.64, SD 1.59 years) and 99 parents (mean age 47.79, SD 6.34 years); 82 adolescent-parent dyads were included in the sample. All parents and all but 1 adolescent reported having a smartphone device (ie, either an iPhone or Android). The majority of adolescents (81/103, 78.6%) and parents (69/99, 70%) reported that they were either comfortable or very comfortable with technology. See Table 1 for additional demographic information about the sample.

**Table 1:** Sample demographic information of this survey study conducted from June 2021 to September 2022 among adolescents with chronic pain (n=103) and their parents (n=99) at a pediatric hospital in the Northeast United States.

Characteristics		Adolescents, n (%)		Parents, n (%)
**Gender**				
	Female	79 (76.7)	90 (91)	
	Male	14 (13.6)	9 (9)	
	Other	10 (9.7)	0 (0)	
**Phone used**				
	Android	15 (14.6)	27 (27)	
	iPhone	87 (84.5)	72 (73)	
	None	1 (1)	0 (0)	
**Comfort with technology**				
	Very uncomfortable	14 (13.6)	6 (6)	
	Uncomfortable	0 (0)	0 (0)	
	Somewhat comfortable	8 (7.8)	24 (24)	
	Comfortable	34 (33)	42 (42)	
	Very comfortable	47 (45.6)	27 (27)	
**Highest level of education**				
	Some high school	N/A^a^	1 (1)	
	High school or GED^b^	N/A	11 (11)	
	Associate degree	N/A	6 (6)	
	Some college	N/A	8 (8)	
	Bachelor’s degree	N/A	40 (40)	
	Master’s degree	N/A	25 (25)	
	Professional or doctoral degree	N/A	8 (8)	

^a^N/A: not applicable.

^b^GED: General Educational Development.

### Adolescent and Parent Endorsement of Passive Data Stream Use

All adolescents and parents provided responses regarding endorsement of passive data stream use. Adolescent endorsement of passive data streams in clinical practice varied by stream and ranged from 35% (36/103; ie, keyboard and microphone) to 75.7% (78/103; ie, accelerometer). Only apps (49/103, 47.6%), keyboard (36/103, 35%), and microphone (36/103, 35%) received less than 50% endorsement. Adolescent endorsement for the use of passive data streams in research also varied by stream and ranged from 37.9% (39/103; ie, microphone) to 74.8% (77/103; ie, accelerometer) of adolescents reporting endorsement. Only keyboard (43/103, 41.7%) and microphone (39/103, 37.9%) received less than 50% endorsement.

Parent endorsement of passive data stream use in clinical practice similarly varied by stream and ranged from 54% (53/99; ie, microphone) to 82% (81/99; ie, accelerometer and screen). Parent endorsement for the use of passive data streams in research ranged from 53% (52/99; ie, microphone) to 83% (82/99; ie, accelerometer). Results of Mann-Whitney nonparametric tests of differences revealed significant differences between adolescents and parents for endorsement of apps (*P*=.02) and keyboard (*P*=.04) for clinical use; no significant differences emerged between adolescents and parents for endorsement of each passive data stream for research purposes. Tables 2 and 3 present the percentages of adolescents and parents who endorsed the use of each stream in clinical practice and research.

Adolescent endorsement for sharing their passively collected data with their parents varied by streams and ranged from 27.2% (28/103) for social activity (ie, Bluetooth, SMS text message and call logs, keyboard, and microphone) to 72.8% (75/103) for activity levels (ie, accelerometer). For phone usage (ie, apps), 39.8% (41/103) of adolescents provided endorsement for sharing their data with their parents; for sleep information (ie, light and screen), 47.6% (49/103) provided such endorsement; and for location (ie, GPS), 53.4% (55/103) provided such endorsement.

Dyadic agreement and disagreement for the endorsement of each passive data stream varied across streams for clinical practice and research use. Dyadic disagreement was greatest for apps (33/82, 40% disagreement for clinical use and 30/82, 37% disagreement for research), keyboard (29/82, 35% disagreement for clinical use and 31/82, 38% disagreement for research), and microphone (31/82, 38% disagreement for clinical use and 30/82, 37% disagreement for research). For passive data streams with the greatest dyadic disagreement, adolescent endorsement within disagreeing dyads was as follows: for apps, 24% (8/33) endorsed use for clinical and 30% (9/30) endorsed use for research; for keyboard, 24% (7/29) endorsed use for clinical and 29% (9/31) endorsed use for research; for microphone, 36% (11/31) endorsed use for clinical and 37% (11/30) endorsed use for research. See Figure S1 in Multimedia Appendix 2 for additional information about the dyadic endorsement of each stream.

**Table 2:** Adolescent and parent endorsement (yes or no) of the use of each passive data stream in clinical practice.

	Adolescents (n=103), n (%)	Parents (n=99), n (%)	*P* value
Accelerometer	78 (75.7)	81 (82)	.44
Apps	49 (47.8)	68 (69)	.02
Bluetooth	65 (63.1)	62 (63)	.36
SMS text and call log	58 (56.3)	65 (66)	.59
Keyboard	36 (35)	54 (55)	.04
Microphone	36 (35)	53 (54)	.34
Light	75 (72.8)	79 (80)	.94
Screen	74 (71.8)	81 (82)	.41
GPS	59 (57.3)	61 (62)	.44

**Table 3:** Adolescent and parent endorsement (yes or no) of the use of each passive data stream in research.

	Adolescents (n=103), n (%)	Parents (n=99), n (%)	*P* value
Accelerometer	77 (74.8)	82 (83)	.44
Apps	55 (53.4)	69 (70)	.15
Bluetooth	66 (64.1)	62 (63)	.36
SMS text and call log	60 (58.3)	64 (65)	.94
Keyboard	43 (41.7)	57 (58)	.07
Microphone	39 (37.9)	52 (53)	.42
Light	74 (71.8)	79 (80)	.96
Screen	72 (69.9)	78 (79)	.57
GPS	63 (61.2)	60 (61)	.27

### Perceived Utility

**Figure 1:**
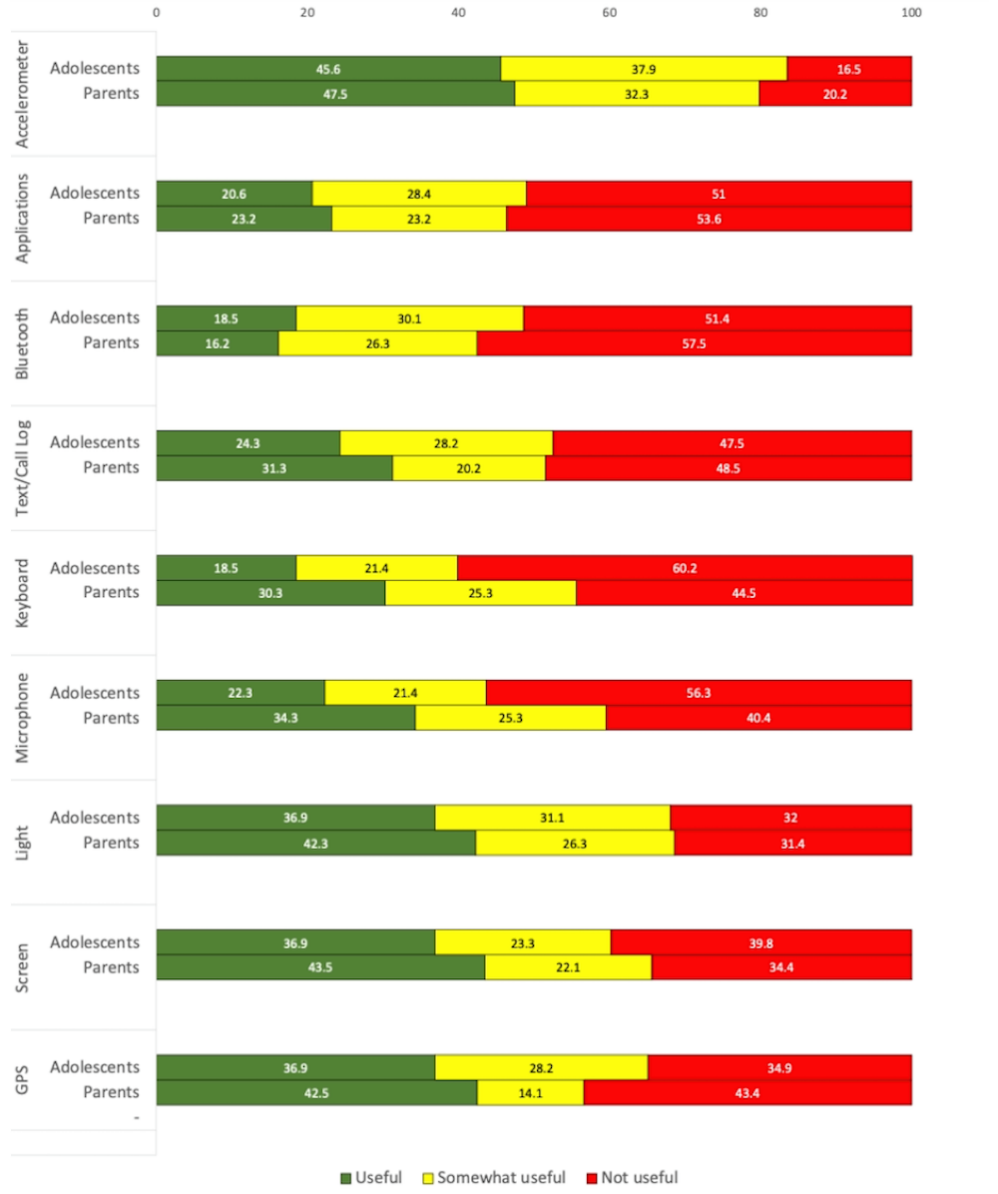
Percentages of adolescent and parent perceived utility by passive data stream.

All adolescents and parents provided responses regarding perceived utility. Figure 1 presents the percentages of adolescents and parents who perceived each passive data stream to be useful (defined as useful or very useful), somewhat useful, or not useful (defined as not useful at all or a little useful). Adolescents and parents alike identified accelerometer, light, screen, and GPS as the streams with the highest utility (ie, greatest percentage of participants considered streams useful or very useful). Similarly, adolescents and parents alike identified apps, Bluetooth, SMS text message and call logs, keyboard, and microphone as the streams with the least utility (ie, greatest number of participants considered streams not useful at all or a little useful). Results from Fisher exact tests of probability indicated no significant differences between adolescents and parents in terms of perceived utility for each passive data stream (*P*_s_>.3). See Figure S2 in Multimedia Appendix 2 for perceived utility for each passive data stream.

### Utility Predicting Endorsement

For adolescents, logistic regression results indicated that the perceived utility of each stream significantly predicted the likelihood of endorsement of its use in both clinical practice (odds ratios [ORs] ranging from 2.90, 95% CI 1.77-4.76 to 4.95, 95% CI 2.34-10.48) and research (ORs ranging from 2.18, 95% CI 1.45-3.27 to 3.80, 95% CI 2.25-6.42), controlling for demographic variables (ie, age and gender). For parents, the same pattern of significance emerged: the perceived utility of each stream significantly predicted the likelihood of endorsement of its use in both clinical practice (ORs ranging from 2.68, 95% CI 1.72-4.19 to 6.91, 95% CI 2.90-16.41) and research (ORs ranging from 1.86, 95% CI 1.22-2.89 to 7.47, 95% CI 2.98-18.71), controlling for demographic variables (ie, age, gender, and education). See Section VI in Multimedia Appendix 2 for full logistic regression results predicting endorsement of each passive data stream.

Pairwise intraclass correlation coefficient results indicated significant (*P*_s_<.001) dyadic interdependence for all primary outcomes of endorsement (ρ ranging from 0.17 to 0.49) with the exception of adolescent and parent endorsement of apps for clinical practice (*P*=.95) and for research (*P*=.95), and adolescent and parent endorsement of GPS for research (*P*=.14). Multilevel logistic regression results indicated that, at the level of the dyad, the perceived utility of each stream predicted the likelihood of endorsement of such streams for clinical (ORs ranging from 2.73, 95% CI 1.51-4.96 to 5.72, 95% CI 2.51-11.06) and for research use (ORs ranging from 1.89, 95% CI 1.16-3.09 to 5.08, 95% CI 2.49-10.36). No significant within-dyad differences predicting endorsement emerged. Similarly, no significant within-dyadic differences in perceived utility predicting endorsement emerged.

### Perceived Concerns

The nature of the concerns varied for each passive data stream in question. For the accelerometer, adolescents and parents endorsed primary concerns with this stream missing activity due to not carrying phones at all times. Adolescents and parents, however, did not endorse significant concern about having their activity data shared. For apps, adolescents and parents reported primary concerns about data inaccuracies due to apps being on in the background; adolescents also endorsed significant concern, more so than parents, about having app usage shared with doctors. For Bluetooth, adolescents and parents endorsed less concern about sharing data from this stream with doctors than they did about this data being seen by people other than doctors. They also endorsed high levels of concern for this stream collecting inaccurate data due to phones not being carried at all times. For SMS text message and call logs, most adolescents and many parents indicated primary concern that they do not call people very often, so data from this stream would not be useful. Of note, over a third of adolescents also indicated concern that they use other apps, other than SMS text messages, to communicate. For light, a majority of adolescents (73/103, 70.9%) and parents (54/99, 55%) indicated primary concerns with data inaccuracies from this stream due to carrying phones in pockets or bags. For screen, adolescents and parents endorsed some concerns about data inaccuracies from this stream, though they did not indicate significant concern about having information from this stream shared with doctors.

For Keyboard, the majority of adolescents (65/103, 63.1%) reported primary concern with data from this stream being shared with their parents. Similarly, a majority of parents (61/99, 62%) endorsed primary concerns about their children sharing this information with them. A majority of adolescents (71/103, 68.9%) also indicated they would not be comfortable with their doctor potentially knowing what they type. Of note, only 19% (13/68) of adolescents and 21% (10/48) of parents endorsed agreement with having typing speed analyzed and keyboard data deleted daily. For microphone, a majority of adolescents (80/103, 77.6%) and parents (69/99, 70%) endorsed primary concerns with their phones picking up everything said in close proximity. A majority of adolescents (70/103, 68%) and parents (57/99, 58%) indicated that it would be difficult for this stream to pick up what is said due to phones being in pockets or bags. In addition, a majority of adolescents (65/103, 63.1%) and most parents (47/99, 48%) also endorsed concerns about sharing this information with their doctor. Only 18% (15/82) of adolescents endorsed agreement with having their voice characteristics analyzed and microphone data deleted daily, whereas 36% (24/66) of parents endorsed agreement with this. Finally, for GPS, adolescents and parents endorsed greater concern about this information being shared with people other than doctors than about the accuracy of the data collected from this stream. Of note, only 11% (5/46) of adolescents and 20% (8/41) of parents endorsed agreement with having GPS data analyzed for places of interest and deleted daily, though 30% (14/46) of adolescents and 29% (12/41) of parents endorsed agreement with having transformed (ie, nonexact) GPS data analyzed. See Figures S4-S12 in Multimedia Appendix 2 for the percentages of adolescents and parents who endorsed specific concerns for each stream.

### Thematic Analysis

Participants provided a total of 157 (79 adolescent responses and 78 parent responses) open-ended responses about additional concerns they had for each passive data stream. Across all streams, 5 overarching themes emerged (ie, privacy concerns, data concerns, absence of personal voice, iatrogenic effects, and other). Privacy concerns referred primarily to participant concerns about the confidentiality of their personal information; data concerns centered around concerns about the accuracy and validity of collected data. Absence of personal voice referred to concerns about data collection dismissing an individual’s thoughts and feelings. Iatrogenic effects captured concerns about unintended negative effects of data collection on patients and their families, and others referred to nonspecific concerns about digital phenotyping methodology in general.

**Figure 2:**
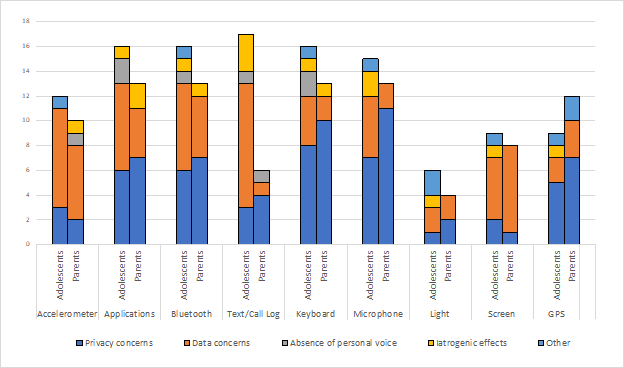
Adolescents’ and parents’ open-response additional concerns by theme.

Some responses were lengthy and contained multiple elements, each representing a specific theme. Figure 2 presents the types of adolescent and parent concerns reported for each theme by the passive data stream. The most common themes of concern that emerged across all streams were privacy concerns and data concerns. Passive data streams with the greatest number of total concerns were apps, Bluetooth, call and SMS text message logs, keyboard, and microphone. Table 4 presents sample quoted responses and quoted response excerpts that were representative of each theme. All personal identifiers were removed from the study data, though presented quoted data are annotated with “adolescent” or “parent” and the specific stream referenced in each open response.

**Table 4:** Quoted samples and excerpts of adolescent and parent open-response additional concerns by theme.

Theme	Adolescent	Parent
Privacy concerns	“The benefits of the information for doctor/researchers does not outweigh the cost of the extreme invasion of privacy.” [female adolescent] “If a physician said to me ‘show me your call logs and activity on your phone for all your apps’ I would think they were untrustworthy and/or up to something. It’s so invasive.” [female adolescent]	“Again, for me as a parent, it is the privacy issue of tracking so much of a person’s life.” [mother] “I am uncomfortable with any app that tracks every single thing that my child does. Unfortunately, scary world and I wouldn’t give permission for any of these apps to be knowingly used.” [mother]
Data concerns	“I am also concerned that my activity is not always a reflection of how I am feeling and functioning.” [female adolescent] “It might give my doctor the wrong idea. For example, I push through a lot of my pain, so just because I am somewhere doesn’t mean I am symptom-free and having a great time. I could be out at restaurants or other venues but spend a lot of time sick in the bathroom. I could be at school but down with the nurse.” [female adolescent]	“My child may leave phone on at night which a music or TV app running to help sleep, but they aren’t actually awake while the phone is left on. It wouldn’t be an accurate measure.” [mother] “Accuracy. I wouldn’t trust the data fully. I wouldn’t want any real treatment plan nor medication prescribed to my any of my kids from using data on an app.” [mother]
Absence of personal voice	“This takes a certain level of trust away from the patient [and] I feel like that level of trust being taken away makes it seem like the patient is unreliable.” [female adolescent] “How about Drs/patient just having a conversation!” [male adolescent]	“I’m not sure how comfortable I am with allowing that much data access when physicians and teams could work with patients to discuss these same issues and usages vs tracking personal data.” [mother] “Would there be somewhere for my child to also record how they were feeling at the time of the activity?” [mother]
Iatrogenic effects	“This would simply teach people to not share when they are feeling upset because they know it is being listened to. This would have a negative impact on the patients over time.” [female adolescent] “I know I would have anxiety over not hanging around a lot of people and then panic over my mental health team judging me or panicky about the fact of how my data is to score how awful my social life is (this is very depressing). Despite the fact I am honest about now having friends with my care team.” [female adolescent]	“My child would consider this a complete invasion of privacy, and it would cause lots of arguments between us.” [mother] “Adding another app like this one would cause huge problems in our household.” [mother] “I’m afraid my child would strongly oppose having applications usage tracked and shared. I think it would alter her usage if she felt ashamed about being on social media too much.” [mother]
Other	“Sensors like these could cause your phones battery to lose power significantly more than it would when your phone is not checking and sending that much data.” [female adolescent]	“This feature will likely be very draining on the phone battery.” [mother]

## Discussion

This study investigated adolescent and parent perspectives on endorsement, perceived utility, and concerns related to passive data stream collection through smartphones for digital phenotyping for clinical and research purposes in youths with chronic pain. Consistent with our hypotheses, results indicated that adolescents and parents generally endorse the use of digital phenotyping and that perceived utility predicts this endorsement. Concerns do exist, however, about privacy and data accuracy. Below, we discuss these results further and suggest their potential implications for clinical practice and future research for a pediatric population with chronic pain.

On the whole, adolescents and parents reported a moderate level of endorsement for their data collected by passive streams being used for clinical and research purposes. Our data suggest a notable level of parental comfort with a digital phenotyping approach to their child’s care using all 9 passive data streams for clinical or research purposes. For adolescents, however, the results were more nuanced and revealed greater stream-specific variability. That is, some streams garnered near universal endorsement from adolescents (ie, accelerometer, screen, and light), while others earned endorsement from only about a third of adolescents (ie, keyboard and microphone). In other words, just because data can be collected through certain smartphone passive data streams does not indicate that adolescents with chronic pain endorse such data collection.

Understanding the types [28] of smartphone-collected data that adolescents with chronic pain are most comfortable with is integral to leveraging digital phenotyping methodology for this population. For example, social activity may be gleaned from Bluetooth, SMS text message and call logs, keyboard, microphone, and GPS data to indicate who someone is with, the content and tenor of their interactions, and even where they are for these interactions [29]. Similarly, apps, keyboard, and screen can be used to assess an individual’s mood and psychological functioning [30] by providing data about how someone feels, what they are spending their time doing, and what they are typing. Further, a different cluster of accelerometer, light, and screen passive data streams may best capture an individual’s sleep through data that indicates when a person is moving or still, in a dark or light room, and on or off their phone [31]. Results from this study showed that passive data streams targeting adolescents’ social activity, mood, and psychological functioning are perceived as much less acceptable than those assessing sleep or exercise. Importantly, this differential perceived acceptability may suggest that greater education or demonstration of benefits for certain clusters of passive data streams may be warranted to gain increased endorsement for use. Similarly, lower reports of perceived acceptability may indicate that better methods of data security and privacy are necessary before such streams are endorsed for use. As such, passive data streams that received lower perceived acceptability in this study need not be blanketly dismissed from future avenues of work, and specific utilities of certain passive data streams should be emphasized for identified populations of interest while still balancing the influence that survey wording may have on risk perception.

These results have critical implications for how digital phenotyping can be used in the care of this patient population. Specifically, for adolescents with chronic pain, a population with marked sleep disturbance [32,33], the high likelihood of endorsement of sleep-related passive data streams (ie, accelerometer, screen, and light) may position this cluster as a particularly promising avenue for accessing otherwise difficult-to-report data. In addition, given the interrelation of functional disability and prognosis [34], tracking participant’s activity levels through an accelerometer may be particularly important for understanding the functioning of patients with chronic pain as compared to their healthy counterparts. The use of wearable devices may also hold critical promise, as may other ways to passively collect such data [35]. By contrast, the reticence of youths in our sample to share aspects of their social activity through smartphone passive data streams may indicate that Bluetooth, SMS text message and call logs, keyboard, microphone, and GPS are a less fruitful cluster of streams to explore. Similarly, the adolescents in this study reported a notably lower likelihood of endorsement for streams targeting their mood and psychological functioning. For youths with chronic pain, anxiety and low mood can be correlates, predictors, and consequences of pain [36-39]. As such, we note that other, more traditional methods of querying this important psychological information may be more preferable than passive data stream collection for our sample of youths with chronic pain. In a similar vein, we also emphasize that our findings are most relevant for our specific population (eg, pediatric pain), and therefore we underscore the importance of identifying uses of specific passive data streams for specific populations of interest (eg, app or keyboard data for depression or suicidality prediction).

For adolescents and parents alike, the quantitative and qualitative findings from this study also suggest 2 major factors contribute to participants’ endorsement of data collection: perceived utility and privacy concerns. Specifically, a certain level of utility inherent to the data must be demonstrated, and privacy concerns must be minimized. Across all streams, participants’ perceived utility predicted their endorsement, and this was true for adolescents, parents, and adolescent-parent dyads. For example, accelerometer, screen, and light were among the streams with the highest levels of endorsement and were also among the streams deemed most useful. This trend is also reflected in our investigation of stream acceptability by its utility. Indeed, so long as streams were deemed at least somewhat useful, they typically received majority endorsement. Privacy concerns, however, also emerged as a critical factor for participants. The 2 streams that did not gain majority endorsement when deemed somewhat useful were keyboard and microphone, the 2 streams that received the greatest number of privacy concerns. These results suggest that even if participants can appreciate the utility of passive stream–collected data for digital phenotyping, privacy concerns must be adequately addressed by clinicians and researchers in order to appropriately implement this technology. Importantly, adolescents also varied in the extent to which they were willing to agree to have their data shared with their parents, which ranged depending on the cluster of passive data streams in question. Consistent with the number of reported privacy concerns, the fewest adolescents provided endorsement for social activity streams (eg, keyboard and microphone), whereas the most adolescents provided endorsement for activity level streams being shared with their parents. Taken together, these findings highlight adolescent privacy concerns in familial, clinical, and research contexts, which may be important considerations for adolescent agreement to participate in other data-sharing opportunities, such as data donation.

In addition to privacy concerns, participants also voiced significant data-specific concerns related to both the accuracy of the data as well as the data’s validity and meaning. In other words, participants want their data to correctly reflect how they are feeling. Adolescents and parents alike conveyed these concerns across all streams, from misgivings that their data would not correctly capture the targeted behavior (eg, if an individual plays a sport but does not carry their phone) to apprehension that their data would actually reveal their true experience (eg, if an individual is active but still in significant pain). Relatedly, participants also voiced a desire for an opportunity to contextualize their data with their experience. For youths with chronic pain and their parents, this desire to explain and “be heard” may be particularly important given their long medical journeys [40], experience of diagnostic uncertainty [41], and belief that providers are misunderstanding their pain [42]. As such, we suggest these considerations be at the forefront of clinical and research use for youths with chronic pain so that the technology of digital phenotyping can be leveraged while still balancing and honoring participant preference to contextualize their data with their own perceived experience. It will be important for clinicians to collaboratively review with their patients the data collected using mobile health tools in order to understand the context of the information gathered and to make sure patients have the opportunity to comment on their data. We note that smartphones may again be leveraged toward this goal through the augmentation of passive data stream collection with smartphone-delivered self-report surveys. Future work may benefit from exploring the inclusion of participant voice alongside passive data collection in this way.

This study also has several methodological limitations that suggest avenues for future research. First, this study investigated adolescent and parent attitudes in a specific population of English-speaking, treatment-seeking youths with chronic pain at 1 hospital in the northeast. As is typical of research in this population, our sample was skewed heavily female for both adolescents [36] and parents [43]. While representative of the population with chronic pain seen in our hospital, our findings should be generalized with caution to other, more diverse populations of youths, and we suggest that future research seek to similarly explore adolescent and parent attitudes toward digital phenotyping in other patient populations or samples of interest, particularly with options for translated versions of survey questions. In addition, our study was limited in its survey methodology, such that participants could only convey their thoughts and feelings through web-based survey responses, mostly in the form of multiple-choice response formats. Although we provided some options for free responses, future work may best capture the nuance and detail of youths’ perspectives through interview-based data collection or focus groups, particularly those that probe the specific dyadic influence on adolescents and parent perspectives. Similarly, although our survey materials aimed to provide objective, unbiased information regarding each data stream and questions that might arise from their use, we acknowledge that we could not exhaustively anticipate all potential questions and concerns from participants. As such, we recommend future work implement more open-ended and interview-style interaction between researchers and participants to minimize the risk of potentially “leading” information or questions. Finally, although our study provides important information about digital phenotyping perspectives, our results do not indicate that these perspectives would remain constant when participants actually have their data collected through smartphone passive data streams. Future studies should begin to pilot and seek feedback during and after actual use of digital phenotyping methods to best understand participant endorsement as well as any concerns that unfold through engagement with these methods. Relatedly, such future studies must also consider participant preferences in the context of ethical implementation of this methodology (eg, consenting and sharing results with participants), which was beyond the scope of this survey. Importantly, future studies must also aim to clarify the validity of such data collection alongside previously validated self-report measures (eg, Pediatric Patient Reported Outcomes Measurement Information System [44] and the Functional Disability Inventory [45,46]).

In conclusion, despite these limitations, the results of this study provide valuable adolescent and parent insights about endorsement, perceived utility, and concerns related to digital phenotyping methodology for clinical and research purposes in youths with chronic pain. Although our findings highlight the general endorsement of this methodology across different passive data streams, the results also reveal the importance of maximizing participant perceived utility, minimizing privacy concerns, and finding ways to incorporate participant contextualization of their own data. Ultimately, these findings can help refine digital phenotyping methodology toward an acceptable, feasible, and ethical implementation of real-time symptom monitoring for assessment and intervention in youths with chronic pain.

## Appendix

Multimedia Appendix 1 Complete REDCap Survey. REDCap: Research Electronic Data Capture.

Multimedia Appendix 2 Supplemental tables and figures.

## Abbreviations

BCH: Boston Children’s Hospital
HIPAA: Health Insurance Portability and Accountability Act
IRB: institutional review board
OR: odds ratio
REDCap: Research Electronic Data Capture

## Data Availability

Deidentified data sets generated and analyzed during this study are available from the corresponding author on reasonable request.
