# Supplementary material for: Adolescent and Parent Perspectives on Digital Phenotyping in Youths With Chronic Pain: Cross-Sectional Mixed Methods Survey Study
Source: J Med Internet Res. 2024 Jan 11;26:e47781. doi: 10.2196/47781 (PMC10811597; doi:10.2196/47781)
Supplement: Multimedia Appendix 1 [file jmir_v26i1e47781_app1.pdf]

Using Smartphone Sensors for Clinical Care and Research

Thank you for participating in this study!

We are conducting this research study to understand how adolescent patients with pain conditions and their parents feel and think about the use of data gathered from smartphones being used for clinical care and research purposes. For this purpose, we are asking you to fill out the following web survey, in which we'll collect some basic demographic information and assess your concerns and opinions on the utility of this technology.

Your opinion is very important to us. It will inform how we can best incorporate smartphones in an acceptable and useful fashion in our clinical research as well as in our assessment and treatment of adolescent pain patients. This survey will take approximately 30 minutes to complete. Please answer all the questions in one sitting. You will not be allowed to return to the suvey if you exit early.

Survey date ______________________________

Demographic Questions

Age	______________________________ (Years)
Are you:	◯ A mother<br>◯ A father<br>◯ A guardian<br>◯ A patient
What is your highest level of education?	◯ Some high school coursework completed, but did not graduate or complete a GED<br>◯ High school or equivalent: you've earned a high school diploma or a GED<br>◯ Associate degree (including occupational or academic degrees)<br>◯ Some college coursework completed, but haven't earned a degree<br>◯ Bachelor's degree: you've earned a four year degree (BS, BA, etc.)<br>◯ Master's degree: you've earned a master's level degree (MA, MS, MENG, MSW, MBA, etc.)<br>◯ Professional school or doctorate degree: you've earned a professional or doctorate level degree (MD, DDC, JD, PhD, EdD, etc.)<br>◯ Other
Please, specify the other education level	______________________________

Familiarity with Techology Question

How comfortable are you with technology?	◯ 1: Very uncomfortable<br>◯ 2: Uncomfortable<br>◯ 3: Somewhat comfortable<br>◯ 4: Comfortable<br>◯ 5: Very comfortable
Your phone is:	◯ Android<br>◯ iPhone<br>◯ Windows phone<br>◯ Other<br>◯ I don't have a phone
Which other phone?	__________________________________

Thank you for filling out the Demographic portion of the survey! Please click on "Submit" and begin the next portion of the survey.

Adolescent Survey

Please complete the survey below.

Thank you!

Introduction

There are multiple sensors built into every smartphone that can be used for many different purposes. In this survey, we would like to ask you about your opinion on having researchers and doctors use information gathered from these sensors in order to better understand how you are doing and treat you. Using short videos, we will explain what the sensors do and provide examples of how they could be used for your treatment and research about your condition. Please watch the videos and fill out the questions for each sensor. There will be a total of 9 sensors presented to you. They are grouped into 5 categories: Activity, Location, Social Activity, Sleep, and Phone Use.

We want to make it clear that we are only interested in your opinion on the usage of these sensors. Your answers to any of these questions will not result in any information being gathered about you. The only purpose of this survey is to understand how patients and their parents feel about the collection of information from phone sensors for treatment and research purposes, which is necessary in order to be able to design future studies in ways that are acceptable and ethical from a patients/parent’s point of view.

Activity Sensors: Accelerometer

Please watch the video and fill out the questions below.

1. Overall how useful do you feel the information from this sensor will be in helping us understand how you are doing?	◯ 1: Not useful at all<br>◯ 2: A little useful<br>◯ 3: Somewhat useful<br>◯ 4: Useful<br>◯ 5: Very useful
2. Would you agree to have your daily activity levels based on this sensor shared with your doctor as part of your treatment plan?	◯ Yes<br>◯ No
3. Would you agree to have your daily activity levels based on this sensor shared with researchers for studies related to your pain condition?	◯ Yes<br>◯ No

5%

4. Please indicate your level of agreement with the following concerns about using this sensor in your treatment plan and/or clinical research from "Strongly Disagree" to "Strongly Agree."

		Strongly disagree	Disagree	Neutral	Agree	Strongly agree
a	I don't think this sensor would be useful for my treatment	◯	◯	◯	◯	◯
b	I don't want anyone to be able to see my activity levels	◯	◯	◯	◯	◯
c	I don't carry my phone with me all the time, so you might miss lots of activity	◯	◯	◯	◯	◯
d	I'm worried that my data might be seen by people other than my doctor	◯	◯	◯	◯	◯

4.2. Do you have any other concerns that were not previously listed?

◯ Yes
◯ No

4.3. Please specify your other concern(s):

____

4.4. How strong is this concern?

◯ Not concerning at all
◯ A little concerning
◯ Somewhat concerning
◯ Concerning
◯ Very concerning

5. Would you be ok with having your activity levels (accelerometer sensor) shared with your parents as part of your medical assessment?

◯ Yes
◯ No

6. Is there anything you think would be important for us to measure to understand your activity levels and how active you are? (Optional)

____

11% Complete

Phone Use Sensors: Applications

Please watch the video and fill out the questions below.

1. Overall, how useful do you feel the information from this sensor will be in helping us understand how you are doing?	◯ 1: Not useful at all<br>◯ 2: A little useful<br>◯ 3: Somewhat useful<br>◯ 4: Useful<br>◯ 5: Very useful
2. Would you agree to this information being shared with your doctor as part of your treatment plan?	◯ Yes<br>◯ No
3. Would you agree to this information being shared with researchers for studies related to your pain condition?	◯ Yes<br>◯ No

16% Complete

4. Please indicate your level of agreement with the following concerns about using this sensor in your treatment plan and/or clinical research from "Strongly Disagree" to "Strongly Agree."

		Strongly disagree	Disagree	Neutral	Agree	Strongly agree
a	I don't think this sensor is useful in treatment	◯	◯	◯	◯	◯
b	I only use my phone to make calls or send and receive text messages, I don't use apps	◯	◯	◯	◯	◯
c	I use a lot of apps at the same time and leave them open in the background. The information about time spent on each app might therefore be wrong	◯	◯	◯	◯	◯
d	I don't feel comfortable with my doctor knowing what apps I use	◯	◯	◯	◯	◯
e	I don't feel comfortable with my doctor knowing how long I use each app	◯	◯	◯	◯	◯

4.2. Do you have any other concerns that were not previously listed?

◯ Yes
◯ No

4.3. Please specify your other concern(s):

____

4.4. How strong is this concern?

◯ 1: Not concerning at all
◯ 2: A little concerning
◯ 3: Neutral
◯ 4: Concerning
◯ 5: Very concerning

5. Would you be ok with having your phone usage (applications sensor) shared with your parents as part of your medical assessment?

◯ Yes
◯ No

6. Is there anything you think would be important for us to measure to understand your phone usage? (Optional)

____

22% Complete

Social Activity Sensors: Bluetooth

Please watch the video and fill out the questions below.

1. Overall, how useful do you feel the information from this sensor will be in helping us understand how you are doing?	◯ 1: Not useful at all<br>◯ 2: A little useful<br>◯ 3: Somewhat useful<br>◯ 4: Useful<br>◯ 5: Very useful
2. Would you agree to this information being shared with your doctor as part of your treatment plan?	◯ Yes<br>◯ No
3. Would you agree to this information being shared with researchers for studies related to your pain condition?	◯ Yes<br>◯ No

27% Complete

4. Please indicate your level of agreement with the following concerns about using this sensor in your treatment plan and/or clinical research from "Strongly Disagree" to "Strongly Agree."

		Strongly disagree	Disagree	Neutral	Agree	Strongly agree
a	I don't think this sensor is useful in treatment	◯	◯	◯	◯	◯
b	I don't carry my phone with me all the time, so the measure won't be accurate	◯	◯	◯	◯	◯
c	I keep my Bluetooth turned off	◯	◯	◯	◯	◯
d	I don't want my doctor to know who I spend time with	◯	◯	◯	◯	◯
e	I am worried that my data might be seen by people other than my doctor	◯	◯	◯	◯	◯

4.2. Do you have any other concerns that were not previously listed?

◯ Yes
◯ No

4.3. Please specify your other concern(s):

____

4.4. How strong is this concern?

◯ 1: Not concerning at all
◯ 2: A little concerning
◯ 3: Neutral
◯ 4: Concerning
◯ 5: Very concerning

33% Complete

Social Activity Sensors: Text Messages and Call Log

Please watch the video and fill out the questions below.

1. Overall, how useful do you feel the information from this sensor will be in helping us understand how you are doing?	◯ 1: Not useful at all<br>◯ 2: A little useful<br>◯ 3: Somewhat useful<br>◯ 4: Useful<br>◯ 5: Very useful
2. Would you agree to this information being shared with your doctor as part of your treatment plan?	◯ Yes<br>◯ No
3. Would you agree to this information being shared with researchers for studies related to your pain condition?	◯ Yes<br>◯ No

38% Complete

Text Message and Call Log

4. Please indicate your level of agreement with the following concerns about using this sensor in your treatment plan and/or clinical research from "Strongly Disagree" to "Strongly Agree."

	Strongly disagree	Disagree	Neutral	Agree	Strongly agree
I don't think this sensor is useful in treatment	◯	◯	◯	◯	◯
I don't use text messages; I use other apps to communicate	◯	◯	◯	◯	◯
I use other apps to call people	◯	◯	◯	◯	◯
I don't call people very often, so this measure would not be useful in treatment	◯	◯	◯	◯	◯
I don't feel comfortable with my doctor having this information	◯	◯	◯	◯	◯

4.2. Do you have any other concerns that were not previously listed?

◯ Yes
◯ No

4.3. Please specify your other concern(s):

____

4.4. How strong is this concern?

◯ 1: Not concerning at all
◯ 2: A little concerning
◯ 3: Neutral
◯ 4: Concerning
◯ 5: Very concerning

44% Complete

Social Activity Sensors: Keyboard

Please watch the video and fill out the questions below.

1. Overall, how useful do you feel the information from this sensor will be in helping us understand how you are doing?	◯ 1: Not useful at all<br>◯ 2: A little useful<br>◯ 3: Somewhat useful<br>◯ 4: Useful<br>◯ 5: Very useful
2. Would you agree to this information being shared with your doctor as part of your treatment plan?	◯ Yes<br>◯ No
3. Would you agree to this information being shared with researchers for studies related to your pain condition?	◯ Yes<br>◯ No

50% Complete

4. Please indicate your level of agreement with the following concerns about using this sensor in your treatment plan and/or clinical research from "Strongly Disagree" to "Strongly Agree."

	Strongly disagree	Disagree	Neutral	Agree	Strongly agree
I don't think this sensor is useful in treatment	◯	◯	◯	◯	◯
I wouldn't want this information to be shared with my parents	◯	◯	◯	◯	◯
I am not comfortable with having my doctor potentially know what I type	◯	◯	◯	◯	◯
I use voice dictation to type, so this measure wouldn't be useful in treatment	◯	◯	◯	◯	◯
I am worried that my data might be seen by people other than my doctor	◯	◯	◯	◯	◯

4.2. Do you have any other concerns that were not previously listed?

◯ Yes
◯ No

4.3. Please specify your other concern(s):

____

4.4. How strong is this concern?

◯ 1: Not concerning at all
◯ 2: A little concerning
◯ 3: Neutral
◯ 4: Concerning
◯ 5: Very concerning

5. If we were to look at your keyboard use in order to analyze your typing speed and deleted all the keyboard data daily, would you feel comfortable about having this information used in your health care?

◯ Yes
◯ No

55% Complete

Social Activity Sensors: Voice

Please watch the video and fill out the questions below.

1. Overall, how useful do you feel the information from this sensor will be in helping us understand how you are doing?	◯ 1: Not useful at all<br>◯ 2: A little useful<br>◯ 3: Somewhat useful<br>◯ 4: Useful<br>◯ 5: Very useful
2. Would you agree to this information being shared with your doctor as part of your treatment plan?	◯ Yes<br>◯ No
3. Would you agree to this information being shared with researchers for studies related to your pain condition?	◯ Yes<br>◯ No

61% Complete

4. Please indicate your level of agreement with the following concerns about using this sensor in your treatment plan and/or clinical research from "Strongly Disagree" to "Strongly Agree."

	Strongly disagree	Disagree	Neutral	Agree	Strongly agree
I don't think this sensor is useful in treatment	◯	◯	◯	◯	◯
I am not comfortable with my phone picking up everything I say in close proximity, even if it can be used for treatment	◯	◯	◯	◯	◯
I use a lot of slang words. It might be difficult for the artificial intelligence to capture what I am saying	◯	◯	◯	◯	◯
I often have my phone in my pocket or bag, so it might be difficult for the sensor to pick up what I say	◯	◯	◯	◯	◯
I am not comfortable with my doctor having this information	◯	◯	◯	◯	◯

4.2. Do you have any other concerns that were not previously listed?

◯ Yes
◯ No

4.3. Please specify your other concern(s):

____

4.4. How strong is this concern?

◯ 1: Not concerning at all
◯ 2: A little concerning
◯ 3: Neutral
◯ 4: Concerning
◯ 5: Very concerning

5. If we were to analyze characteristics of your voice, such as loudness and speaking rate and then deleted the voice data daily, would you feel comfortable about having this information used in your health care?

◯ Yes
◯ No

6. Would you be ok with having your social activity (bluetooth, text message and call log, keyboard, and voice sensors) shared with your parents as part of your medical assessment?

◯ Yes
◯ No

7. Is there anything you think would be important for us to measure to understand your social activity? (Optional)

____

66% Complete

Sleep Sensors: Light

Please watch the video and fill out the questions below.

1. Overall, how useful do you feel the information from this sensor will be in helping us understand how you are doing?	◯ 1: Not useful at all<br>◯ 2: A little useful<br>◯ 3: Somewhat useful<br>◯ 4: Useful<br>◯ 5: Very useful
2. Would you agree to this information being shared with your doctor as part of your treatment plan?	◯ Yes<br>◯ No
3. Would you agree to this information being shared with researchers for studies related to your pain condition?	◯ Yes<br>◯ No

71% Complete

4. Please indicate your level of agreement with the following concerns about using this sensor in your treatment plan and/or clinical research from "Strongly Disagree" to "Strongly Agree."

		Strongly disagree	Disagree	Neutral	Agree	Strongly agree
a	I don't think this sensor is useful in treatment	◯	◯	◯	◯	◯
b	I don't carry my phone with me all the time, so the measure won't be accurate	◯	◯	◯	◯	◯
c	I carry my phone in my pocket or in my bag, so I am not sure if this measure will be accurate	◯	◯	◯	◯	◯
d	I turn my phone off at night, so I am not sure this measure will gather the information you want	◯	◯	◯	◯	◯
e	I turn my phone off when I don't feel well, so I am not sure if this measure will be helpful in my treatment plan	◯	◯	◯	◯	◯

4.2. Do you have any other concerns that were not previously listed?

◯ Yes
◯ No

4.3. Please specify your other concern(s):

____

4.4. How strong is this concern?

◯ 1: Not concerning at all
◯ 2: A little concerning
◯ 3: Neutral
◯ 4: Concerning
◯ 5: Very concerning

77% Complete

Sleep Sensors: Screen

Please watch the video and fill out the questions below.

1. Overall, how useful do you feel the information from this sensor will be in helping us understand how you are doing?	◯ 1: Not useful at all<br>◯ 2: A little useful<br>◯ 3: Somewhat useful<br>◯ 4: Useful<br>◯ 5: Very useful
2. Would you agree to this information being shared with your doctor as part of your treatment plan?	◯ Yes<br>◯ No
3. Would you agree to this information being shared with researchers for studies related to your pain condition?	◯ Yes<br>◯ No

82% Complete

4. Please indicate your level of agreement with the following concerns about using this sensor in your treatment plan and/or clinical research from "Strongly Disagree" to "Strongly Agree."

	Strongly disagree	Disagree	Neutral	Agree	Strongly agree
I don't think this sensor is useful in treatment	○	○	○	○	○
I wouldn't want people to know when I use my phone during the day	○	○	○	○	○
I wouldn't want people to know when I use my phone during the night	○	○	○	○	○
I don't use my phone at night, so the measure won't be accurate	○	○	○	○	○
I don't feel comfortable with my doctor having this information	○	○	○	○	○

4.2. Do you have any other concerns that were not previously listed?

○ Yes
○ No

4.3. Please specify your other concern(s):

____

4.4. How strong is this concern?

○ 1: Not concerning at all
○ 2: A little concerning
○ 3: Neutral
○ 4: Concerning
○ 5: Very concerning

5. Would you be ok with having your sleep information (light and screen sensors) shared with your parents as part of your medical assessment?

○ Yes
○ No

6. Is there anything you think would be important for us to measure to understand your sleep? (Optional)

____

88% Complete

Location Sensors: GPS

Please watch the video and fill out the questions below.

1. Overall, how useful do you feel the information from this sensor will be in helping us understand how you are doing?	◯ 1: Not useful at all<br>◯ 2: A little useful<br>◯ 3: Somewhat useful<br>◯ 4: Useful<br>◯ 5: Very useful
2. Would you agree to this information being shared with your doctor as part of your treatment plan?	◯ Yes<br>◯ No
3. Would you agree to this information being shared with researchers for studies related to your pain condition?	◯ Yes<br>◯ No

93% Complete

4. Please indicate your level of agreement with the following concerns about using this sensor in your treatment plan and/or clinical research from "Strongly Disagree" to "Strongly Agree."

	Strongly disagree	Disagree	Neutral	Agree	Strongly agree
I don't think this sensor is useful in treatment	◯	◯	◯	◯	◯
When I leave home, I don't usually carry my phone with me	◯	◯	◯	◯	◯
I am not comfortable about the clinician knowing where I spend my time during the day	◯	◯	◯	◯	◯
I am worried that my data might be seen by people other than my doctor	◯	◯	◯	◯	◯
I turn my phone off at times during the day, so this measure would not be helpful	◯	◯	◯	◯	◯

4.2. Do you have any other concerns that were not previously listed?

◯ Yes
◯ No

4.3. Please specify your other concern(s):

____

4.4. How strong is this concern?

◯ 1: Not concerning at all
◯ 2: A little concerning
◯ 3: Neutral
◯ 4: Concerning
◯ 5: Very concerning

5. If we use your GPS data to tell us how much time you spend at select places of interest, such as home and school and then delete it daily, would you feel comfortable about having this information used in your health care?

◯ Yes
◯ No

6. If your phone was to provide transformed GPS data, which would not show exactly where you are but could let us know how much time you spend at various locations during the day and how much you move around, would you feel comfortable about having this information used in your health care?

◯ Yes
◯ No

7. Would you be ok with having where you spend time (GPS sensor) shared with your parents as part of your medical assessment?

◯ Yes
◯ No

8. Is there anything you think would be important for us to measure to understand your location? (Optional)

____

100% Complete

Parent Survey

Please complete the survey below.

Thank you!

Introduction

There are multiple sensors built into every smartphone that can be used for many different purposes. In this survey, we would like to ask you about your opinion on having researchers and doctors use information gathered from these sensors in order to better understand how your child is doing. Using short videos, we will explain what the sensors do and provide examples of how they could be used for your child's treatment and research about your child's condition. Please watch the videos and fill out the questions for each sensor. There will be a total of 9 sensors presented to you. They are grouped into 5 categories: Activity, Location, Social Activity, Sleep, and Phone Use.

We want to make it clear that we are only interested in your opinion on the usage of these sensors. Your answers to any of these questions will not result in any information being gathered about you. The only purpose of this survey is to understand how patients and their parents feel about the collection of information from phone sensors for treatment and research purposes, which is necessary in order to be able to design future studies in ways that are acceptable and ethical from a patients/parent's point of view.

Activity Sensors: Accelerometer

Please watch the video and fill out the questions below.

1. Overall how useful do you feel the information from this sensor will be in helping us understand how your child is doing?	◯ 1: Not useful at all<br>◯ 2: A little useful<br>◯ 3: Somewhat useful<br>◯ 4: Useful<br>◯ 5: Very useful
2. Would you agree to have your child's daily activity levels based on this sensor shared with your child's doctor as part of their treatment plan?	◯ Yes<br>◯ No
3. Would you agree to have your child's daily activity levels based on this sensor shared with researchers for studies related to your child's pain condition?	◯ Yes<br>◯ No

5%

4. Please indicate your level of agreement with the following concerns about using this sensor in your child's treatment plan and/or clinical research from "Strongly Disagree" to "Strongly Agree."

		Strongly disagree	Disagree	Neutral	Agree	Strongly agree
a	I don't think this sensor would be useful for my child's treatment	◯	◯	◯	◯	◯
b	I don't want anyone to be able to see my child's activity levels	◯	◯	◯	◯	◯
c	My child doesn't carry their phone with them all the time, so you might miss lots of activity	◯	◯	◯	◯	◯
d	I'm worried that my child's data might be seen by people other than my child's doctor	◯	◯	◯	◯	◯

4.2. Do you have any other concerns that were not previously listed?

◯ Yes
◯ No

4.3. Please specify your other concern(s):

____

4.4. How strong is this concern?

◯ 1: Not concerning at all
◯ 2: A little concerning
◯ 3: Neutral
◯ 4: Concerning
◯ 5: Very concerning

5. Is there anything you think would be important for us to measure to understand your child's activity levels (accelerometer sensor) and how active they are? (Optional)

____

11% Complete

Phone Usage Sensors: Applications

Please watch the video and fill out the questions below.

1. Overall, how useful do you feel the information from this sensor will be in helping us understand how your child is doing?	◯ 1: Not useful at all<br>◯ 2: A little useful<br>◯ 3: Somewhat useful<br>◯ 4: Useful<br>◯ 5: Very useful
2. Would you agree to this information being shared with your child's doctor as part of your child's treatment plan?	◯ Yes<br>◯ No
3. Would you agree to this information being shared with researchers for studies related to your child's pain condition?	◯ Yes<br>◯ No

16% Complete

4. Please indicate your level of agreement with the following concerns about using this sensor in your child's treatment plan and/or clinical research from "Strongly Disagree" to "Strongly Agree."

		Strongly disagree	Disagree	Neutral	Agree	Strongly agree
a	I don't think this sensor is useful in treatment	○	○	○	○	○
b	My child only uses their phone to make calls or send and receive text messages, they don't use apps	○	○	○	○	○
c	My child uses a lot of apps at the same time and leaves them open in the background. The information about time spent on each app might therefore be wrong	○	○	○	○	○
d	I don't feel comfortable with my child's doctor knowing what apps my child uses	○	○	○	○	○
e	I don't feel comfortable with my child's doctor knowing how long my child uses each app	○	○	○	○	○

4.2. Do you have any other concerns that were not previously listed?

○ Yes
○ No

4.3. Please specify your other concern(s):

____

4.4. How strong is this concern?

○ 1: Not concerning at all
○ 2: A little concerning
○ 3: Neutral
○ 4: Concerning
○ 5: Very concerning

5. Is there anything you think would be important for us to measure to understand your child's phone usage (applications sensor)? (Optional)

____

22% Complete

Social Activity Sensors: Bluetooth

Please watch the video and fill out the questions below.

1. Overall, how useful do you feel the information from this sensor will be in helping us understand how your child is doing?	◯ 1: Not useful at all<br>◯ 2: A little useful<br>◯ 3: Somewhat useful<br>◯ 4: Useful<br>◯ 5: Very useful
2. Would you agree to this information being shared with your child's doctor as part of your child's treatment plan?	◯ Yes<br>◯ No
3. Would you agree to this information being shared with researchers for studies related to your child's pain condition?	◯ Yes<br>◯ No

27% Complete

4. Please indicate your level of agreement with the following concerns about using this sensor in your child's treatment plan and/or clinical research from "Strongly Disagree" to "Strongly Agree."

		Strongly disagree	Disagree	Neutral	Agree	Strongly agree
a	I don't think this sensor is useful in treatment	◯	◯	◯	◯	◯
b	My child doesn't carry their phone with them all the time, so the measure won't be accurate	◯	◯	◯	◯	◯
c	My child keeps their Bluetooth turned off	◯	◯	◯	◯	◯
d	I don't want my child's doctor to know who they spend time with	◯	◯	◯	◯	◯
e	I am worried that my child's data might be seen by people other than my child's doctor	◯	◯	◯	◯	◯

4.2. Do you have any other concerns that were not previously listed?

◯ Yes
◯ No

4.3. Please specify your other concern(s):

____

4.4. How strong is this concern?

◯ 1: Not concerning at all
◯ 2: A little concerning
◯ 3: Neutral
◯ 4: Concerning
◯ 5: Very concerning

33% Complete

Social Activity Sensors: Text Messages and Call Log

Please watch the video and fill out the questions below.

1. Overall, how useful do you feel the information from this sensor will be in helping us understand how your child is doing?	◯ 1: Not useful at all<br>◯ 2: A little useful<br>◯ 3: Somewhat useful<br>◯ 4: Useful<br>◯ 5: Very useful
2. Would you agree to this information being shared with your child's doctor as part of your child's treatment plan?	◯ Yes<br>◯ No
3. Would you agree to this information being shared with researchers for studies related to your child's pain condition?	◯ Yes<br>◯ No

38% Complete

4. Please indicate your level of agreement with the following concerns about using this sensor in your child's treatment plan and/or clinical research from "Strongly Disagree" to "Strongly Agree."

	Strongly disagree	Disagree	Neutral	Agree	Strongly agree
I don't think this sensor is useful in treatment	◯	◯	◯	◯	◯
My child doesn't use text messages; they use other apps to communicate	◯	◯	◯	◯	◯
My child uses other apps to call people	◯	◯	◯	◯	◯
My child doesn't call people very often, so this measure would not be useful in treatment	◯	◯	◯	◯	◯
I don't feel comfortable with my child's doctor having this information	◯	◯	◯	◯	◯

4.2. Do you have any other concerns that were not previously listed?

◯ Yes
◯ No

4.3. Please specify your other concern(s):

____

4.4. How strong is this concern?

◯ 1: Not concerning at all
◯ 2: A little concerning
◯ 3: Neutral
◯ 4: Concerning
◯ 5: Very concerning

44% Complete

Social Activity Sensors: Keyboard

Please watch the video and fill out the questions below.

1. Overall, how useful do you feel the information from this sensor will be in helping us understand how your child is doing?	◯ 1: Not useful at all<br>◯ 2: A little useful<br>◯ 3: Somewhat useful<br>◯ 4: Useful<br>◯ 5: Very useful
2. Would you agree to this information being shared with your child's doctor as part of your child's treatment plan?	◯ Yes<br>◯ No
3. Would you agree to this information being shared with researchers for studies related to your child's pain condition?	◯ Yes<br>◯ No

50% Complete

4. Please indicate your level of agreement with the following concerns about using this sensor in your child's treatment plan and/or clinical research from "Strongly Disagree" to "Strongly Agree."

	Strongly disagree	Disagree	Neutral	Agree	Strongly agree
I don't think this sensor is useful in treatment	◯	◯	◯	◯	◯
My child wouldn't want this information to be shared with their parents	◯	◯	◯	◯	◯
I am not comfortable with having my child's doctor potentially know what I type	◯	◯	◯	◯	◯
My child uses voice dictation to type, so this measure wouldn't be useful in treatment	◯	◯	◯	◯	◯
I am worried that my child's data might be seen by people other than my child's doctor	◯	◯	◯	◯	◯

4.2. Do you have any other concerns that were not previously listed?

◯ Yes
◯ No

4.3. Please specify your other concern(s): __________________________________

4.4. How strong is this concern?

◯ 1: Not concerning at all
◯ 2: A little concerning
◯ 3: Neutral
◯ 4: Concerning
◯ 5: Very concerning

5. If we were to look at your child's keyboard use in order to analyze their typing speed and deleted all the keyboard data daily, would you feel comfortable about having this information used in your child's health care?

◯ Yes
◯ No

55% Complete

Social Activity Sensors: Voice

Please watch the video and fill out the questions below.

1. Overall, how useful do you feel the information from this sensor will be in helping us understand how your child is doing?	◯ 1: Not useful at all<br>◯ 2: A little useful<br>◯ 3: Somewhat useful<br>◯ 4: Useful<br>◯ 5: Very useful
2. Would you agree to this information being shared with your child's doctor as part of your child's treatment plan?	◯ Yes<br>◯ No
3. Would you agree to this information being shared with researchers for studies related to your child's pain condition?	◯ Yes<br>◯ No

60% Complete

4. Please indicate your level of agreement with the following concerns about using this sensor in your child's treatment plan and/or clinical research from "Strongly Disagree" to "Strongly Agree."

	Strongly disagree	Disagree	Neutral	Agree	Strongly agree
I don't think this sensor is useful in treatment	◯	◯	◯	◯	◯
I am not comfortable with my child's phone picking up everything they say in close proximity, even if it can be used for treatment	◯	◯	◯	◯	◯
My child uses a lot of slang words. It might be difficult for the artificial intelligence to capture what they are saying	◯	◯	◯	◯	◯
My child often has their phone in their pocket or bag, so it might be difficult for the sensor to pick up what they say	◯	◯	◯	◯	◯
I am not comfortable with my child's doctor having this information	◯	◯	◯	◯	◯

4.2. Do you have any other concerns that were not previously listed?

◯ Yes
◯ No

4.3. Please specify your other concern(s):

____

4.4. How strong is this concern?

◯ 1: Not concerning at all
◯ 2: A little concerning
◯ 3: Neutral
◯ 4: Concerning
◯ 5: Very concerning

5. If we were to analyze characteristics of your child's voice, such as loudness and speaking rate and then deleted the voice data daily, would you feel comfortable about having this information used in your child's health care?

◯ Yes
◯ No

6. Is there anything you think would be important for us to measure to understand your child's social activity (bluetooth, text messages and call log, keyboard, and voice sensors)? (Optional)

____

66% Complete

Screen Sensors: Light

Please watch the video and fill out the questions below.

1. Overall, how useful do you feel the information from this sensor will be in helping us understand how your child is doing?	◯ 1: Not useful at all<br>◯ 2: A little useful<br>◯ 3: Somewhat useful<br>◯ 4: Useful<br>◯ 5: Very useful
2. Would you agree to this information being shared with your child's doctor as part of your child's treatment plan?	◯ Yes<br>◯ No
3. Would you agree to this information being shared with researchers for studies related to your child's pain condition?	◯ Yes<br>◯ No

71% Complete

4. Please indicate your level of agreement with the following concerns about using this sensor in your child's treatment plan and/or clinical research from "Strongly Disagree" to "Strongly Agree."

		Strongly disagree	Disagree	Neutral	Agree	Strongly agree
a	I don't think this sensor is useful in treatment	◯	◯	◯	◯	◯
b	My child doesn't carry their phone with them all the time, so the measure won't be accurate	◯	◯	◯	◯	◯
c	My child carries their phone in their pocket or in their bag, so I am not sure if this measure will be accurate	◯	◯	◯	◯	◯
d	My child turns their phone off at night, so I am not sure this measure will gather the information you want	◯	◯	◯	◯	◯
e	My child turns their phone off when they don't feel well, so I am not sure if this measure will be helpful in my child's treatment plan	◯	◯	◯	◯	◯

4.2. Do you have any other concerns that were not previously listed?

◯ Yes
◯ No

4.3. Please specify your other concern(s):

____

4.4. How strong is this concern?

◯ 1: Not concerning at all
◯ 2: A little concerning
◯ 3: Neutral
◯ 4: Concerning
◯ 5: Very concerning

77% Complete

Sleep Sensors: Screen

Please watch the video and fill out the questions below.

1. Overall, how useful do you feel the information from this sensor will be in helping us understand how your child is doing?	◯ 1: Not useful at all<br>◯ 2: A little useful<br>◯ 3: Somewhat useful<br>◯ 4: Useful<br>◯ 5: Very useful
2. Would you agree to this information being shared with your child's doctor as part of your child's treatment plan?	◯ Yes<br>◯ No
3. Would you agree to this information being shared with researchers for studies related to your child's pain condition?	◯ Yes<br>◯ No

82% Complete

4. Please indicate your level of agreement with the following concerns about using this sensor in your child's treatment plan and/or clinical research from "Strongly Disagree" to "Strongly Agree."

	Strongly disagree	Disagree	Neutral	Agree	Strongly agree
I don't think this sensor is useful in treatment	◯	◯	◯	◯	◯
I wouldn't want people to know when my child uses their phone during the day	◯	◯	◯	◯	◯
I wouldn't want people to know when my child uses their phone during the night	◯	◯	◯	◯	◯
My child doesn't use their phone at night, so the measure won't be accurate	◯	◯	◯	◯	◯
I don't feel comfortable with my child's doctor having this information	◯	◯	◯	◯	◯

4.2. Do you have any other concerns that were not previously listed?

◯ Yes
◯ No

4.3. Please specify your other concern(s): ______________________________

4.4. How strong is this concern?

◯ 1: Not concerning at all
◯ 2: A little concerning
◯ 3: Neutral
◯ 4: Concerning
◯ 5: Very concerning

5. Is there anything you think would be important for us to measure to understand your child's sleep (screen and light sensors)? (Optional) ______________________________

88% Complete

Location Sensors: GPS

Please watch the video and fill out the questions below.

1. Overall, how useful do you feel the information from this sensor will be in helping us understand how your child is doing?	◯ 1: Not useful at all<br>◯ 2: A little useful<br>◯ 3: Somewhat useful<br>◯ 4: Useful<br>◯ 5: Very useful
2. Would you agree to this information being shared with your child's doctor as part of your child's treatment plan?	◯ Yes<br>◯ No
3. Would you agree to this information being shared with researchers for studies related to your child's pain condition?	◯ Yes<br>◯ No

93% Complete

4. Please indicate your level of agreement with the following concerns about using this sensor in your child's treatment plan and/or clinical research from "Strongly Disagree" to "Strongly Agree."

	Strongly disagree	Disagree	Neutral	Agree	Strongly agree
I don't think this sensor is useful in treatment	◯	◯	◯	◯	◯
When my child leaves home, they don't usually carry their phone with them	◯	◯	◯	◯	◯
I am not comfortable about the clinician knowing where my child spends their time during the day	◯	◯	◯	◯	◯
I am worried that my child's data might be seen by people other than my child's doctor	◯	◯	◯	◯	◯
My child turns their phone off at times during the day, so this measure would not be helpful	◯	◯	◯	◯	◯

4.2. Do you have any other concerns that were not previously listed?

◯ Yes
◯ No

4.3. Please specify your other concern(s):

____

4.4. How strong is this concern?

◯ 1: Not concerning at all
◯ 2: A little concerning
◯ 3: Neutral
◯ 4: Concerning
◯ 5: Very concerning

5. If we use your child's GPS data to tell us how much time your child spends at select places of interest, such as home and school and then delete it daily, would you feel comfortable about having this information used in your child's health care?

◯ Yes
◯ No

6. If your child's phone was to provide transformed GPS data, which would not show exactly where your child is but could let us know how much time they spend at various locations during the day and how much they move around, would you feel comfortable about having this information used in your child's health care?

◯ Yes
◯ No

7. Is there anything you think would be important for us to measure to understand your child's location (GPS sensor)? (Optional)

____

100% Complete
