# Supplementary material for: Adolescent and Parent Perspectives on Digital Phenotyping in Youths With Chronic Pain: Cross-Sectional Mixed Methods Survey Study
Source: J Med Internet Res. 2024 Jan 11;26:e47781. doi: 10.2196/47781 (PMC10811597; doi:10.2196/47781)
Supplement: Multimedia Appendix 2 [file jmir_v26i1e47781_app2.docx]

SUPPLEMENTAL INFORMATION

**Table of Contents:**

**I. Codebook for Open Responses**

**II. Dyadic Disagreement for Endorsement of Use of Each Passive Data Stream**

**III. Perceived Utility for Each Passive Data Stream**

**IV. Endorsement by Utility for Each Passive Data Stream**

**V. Additional Concerns for Each Passive Data Stream**

**VI. Logistic Regression Results Predicting Endorsement of Each Passive Data Stream**

**VII. COREQ Checklist**

**VIII. REDCap Survey (see separate attached PDF)**

**I. Codebook for Open Responses**

**Table S1.** *Codebook for Open-Response Concerns*

| **Category** | **Code** | **Description** |
| --- | --- | --- |
| 1 | Privacy Concerns | Participants express privacy concerns |
| 2 | Data Concerns | Participants express concerns about accuracy and validity of data collected (“What would the data mean?”) |
| 3 | Absence of Personal Voice | Participants express concerns that data collection would be generally dismissive of patient’s thoughts/feelings  (“What about me?”) |
| 4 | Iatrogenic Effects | Participants express that data collection could have unintended iatrogenic effects on patients |
| 5 | Other | Participants express other non-specific concerns about digital phenotyping methodology |

**II. Dyadic Disagreement for Endorsement of Use of Each Passive Data Stream**

**Figure S1.** *Dyadic Disagreement for Endorsement (Yes/No) of Use of Each Passive Data Stream for Clinical Practice and Research*

**III. Perceived Utility for Each Passive Data Stream**

**Figure S2.** *Adolescent and Parent Perceived Utility for Each Passive Data Stream*

**IV. Endorsement by Utility for Each Passive Data Stream**

**Figure S3A.** *Adolescent Endorsement by Utility of Accelerometer*

*Parent Endorsement by Utility of Accelerometer*

**Note:** In the above figures, the overlay number indicates the number of participants who endorsed (yes/no) AND reported the indicated level of utility. For example, in Figure 3A, 4 total adolescents reported that accelerometer was not useful at all. Of those 4, 3 endorsed its use (yes), 1 did not (no).

**Figure S3B.** *Adolescent Perceived Acceptability by Utility of Applications*

*Parent Perceived Acceptability by Utility of Applications*

**Figure S3C.** *Adolescent Perceived Acceptability by Utility of Bluetooth*

*Parent Perceived Acceptability by Utility of Bluetooth*

**Figure S3D.** *Adolescent Perceived Acceptability by Utility of Text/Call Log*

*Parent Perceived Acceptability by Utility of Text/Call Log*

**Figure S3E.** *Adolescent Perceived Acceptability by Utility of Keyboard*

*Parent Perceived Acceptability by Utility of Keyboard*

**Figure S3F.** *Adolescent Perceived Acceptability by Utility of Microphone*

*Parent Perceived Acceptability by Utility of Microphone*

**Figure S3G.** *Adolescent Perceived Acceptability by Utility of Light*

*Parent Perceived Acceptability by Utility of Light*

**Figure S3H.** *Adolescent Perceived Acceptability by Utility of Screen*

*Parent Perceived Acceptability by Utility of Screen*

**Figure S3I.** *Adolescent Perceived Acceptability by Utility of GPS*

*Parent Perceived Acceptability by Utility of GPS*

**V. Additional Concerns for Each Passive Data Stream**

**Figure S4.** *Adolescent and Parent Concerns for Accelerometer*

**Figure S5.** *Adolescent and Parent Concerns for Applications*

**Figure S6.** *Adolescent and Parent Concerns for Bluetooth*

**Figure S7.** *Adolescent and Parent Concerns for Text/Call Logs*

**Figure S8.** *Adolescent and Parent Concerns for Keyboard*

**Figure S9.** *Adolescent and Parent Concerns for Microphone*

**Figure S10.** *Adolescent and Parent Concerns for Screen*

**Figure S11.** *Adolescent and Parent Concerns for Light*

**Figure S12.**

*Adolescent and Parent Concerns for GPS*

**VI. Logistic Regression Results Predicting Endorsement of Each Passive Data Stream**

For adolescent regressions displayed below, there were three gender categories (male, female, and other), therefore the reference category for gender is other (Tables S2-S19). For parent regressions displayed below, there were two gender categories (male and female), therefore the reference category for gender is male (Tables S20-S37).

**Table S2.** Logistic Regression Analysis Predicting Adolescent Endorsement of Accelerometer for Clinical Practice

|  |  | | |  |  | |
| --- | --- | --- | --- | --- | --- | --- |
| Predictor | β | *SE* β | Wald’s χ^2^ | *df* | *p* | *e^β^* |
| Constant | -6.30 | 3.62 | 3.03 | 1 | 0.08 | 0.01 |
| Utility | 1.60 | 0.38 | 17.46 | 1 | <0.001 | 4.95 |
| Age | 0.21 | 0.19 | 1.26 | 1 | 0.26 | 1.23 |
| Gender |  |  | 0.85 | 2 | 0.65 |  |
| Female | -0.89 | 1.12 | 0.63 | 1 | 0.43 | 0.41 |
| Male | -0.43 | 1.34 | 0.11 | 1 | 0.75 | 0.65 |
| Model Summary | Log Likelihood | | Cox & Snell *R*^2^ | | Nagelkerke *R*^2^ | |
|  | 83.98 | | 0.25 | | 0.38 | |

**Table S3.** Logistic Regression Analysis Predicting Adolescent Endorsement of Accelerometer for Research Purposes

|  |  | | |  |  | |
| --- | --- | --- | --- | --- | --- | --- |
| Predictor | β | *SE* β | Wald’s χ^2^ | *df* | *p* | *e^β^* |
| Constant | -3.12 | 3.25 | 0.92 | 1 | 0.34 | 0.04 |
| Utility | 1.29 | 0.33 | 15.38 | 1 | <0.001 | 3.63 |
| Age | 0.07 | 0.17 | 0.15 | 1 | 0.70 | 1.07 |
| Gender |  |  | 1.17 | 2 | 0.56 |  |
| Female | -1.03 | 1.11 | 0.86 | 1 | 0.35 | 0.36 |
| Male | -0.52 | 1.32 | 0.15 | 1 | 0.70 | 0.60 |
| Model Summary | Log Likelihood | | Cox & Snell *R*^2^ | | Nagelkerke *R*^2^ | |
|  | 92.23 | | 0.21 | | 0.30 | |

**Table S4.** Logistic Regression Analysis Predicting Adolescent Endorsement of Applications for Clinical Practice

|  |  | | |  |  | |
| --- | --- | --- | --- | --- | --- | --- |
| Predictor | β | *SE* β | Wald’s χ^2^ | *df* | *p* | *e^β^* |
| Constant | -1.60 | 2.55 | 0.39 | 1 | 0.53 | 0.20 |
| Utility | 1.27 | 0.26 | 23.30 | 1 | <0.001 | 3.56 |
| Age | -0.06 | 0.15 | 0.15 | 1 | 0.70 | 0.94 |
| Gender |  |  | 1.23 | 2 | 0.54 |  |
| Female | -0.83 | 0.76 | 1.19 | 1 | 0.28 | 0.44 |
| Male | -0.55 | 0.99 | 0.31 | 1 | 0.58 | 0.58 |
| Model Summary | Log Likelihood | | Cox & Snell *R*^2^ | | Nagelkerke *R*^2^ | |
|  | 104.32 | | 0.30 | | 0.40 | |

**Table S5.** Logistic Regression Analysis Predicting Adolescent Endorsement of Applications for Research Purposes

|  |  | | |  |  | |
| --- | --- | --- | --- | --- | --- | --- |
| Predictor | β | *SE* β | Wald’s χ^2^ | *df* | *p* | *e^β^* |
| Constant | -2.36 | 2.60 | 0.83 | 1 | 0.36 | 0.09 |
| Utility | 1.29 | 0.27 | 23.57 | 1 | <0.001 | 3.62 |
| Age | -0.04 | 0.15 | 0.09 | 1 | 0.77 | 0.96 |
| Gender |  |  | 0.21 | 2 | 0.90 |  |
| Female | 0.11 | 0.75 | 0.02 | 1 | 0.88 | 1.12 |
| Male | 0.42 | 0.99 | 0.18 | 1 | 0.67 | 1.52 |
| Model Summary | Log Likelihood | | Cox & Snell *R*^2^ | | Nagelkerke *R*^2^ | |
|  | 103.91 | | 0.30 | | 0.39 | |

**Table S6.** Logistic Regression Analysis Predicting Adolescent Endorsement of Bluetooth for Clinical Practice

|  |  | | |  |  | |
| --- | --- | --- | --- | --- | --- | --- |
| Predictor | β | *SE* β | Wald’s χ^2^ | *df* | *p* | *e^β^* |
| Constant | -1.96 | 2.51 | 0.61 | 1 | 0.43 | 0.14 |
| Utility | 0.88 | 0.24 | 13.75 | 1 | <0.001 | 2.42 |
| Age | 0.05 | 0.15 | 0.09 | 1 | 0.76 | 1.05 |
| Gender |  |  | 2.40 | 2 | 0.30 |  |
| Female | -0.09 | 0.78 | 0.01 | 1 | 0.91 | 0.91 |
| Male | -1.11 | 0.96 | 1.34 | 1 | 0.25 | 0.33 |
| Model Summary | Log Likelihood | | Cox & Snell *R*^2^ | | Nagelkerke *R*^2^ | |
|  | 112.57 | | 0.19 | | 0.26 | |

**Table S7.** Logistic Regression Analysis Predicting Adolescent Endorsement of Bluetooth for Research Purposes

|  |  | | |  |  | |
| --- | --- | --- | --- | --- | --- | --- |
| Predictor | β | *SE* β | Wald’s χ^2^ | *df* | *p* | *e^β^* |
| Constant | 0.60 | 2.53 | 0.06 | 1 | 0.81 | 1.81 |
| Utility | 0.98 | 0.25 | 14.34 | 1 | <0.001 | 2.53 |
| Age | -0.12 | 0.15 | 0.68 | 1 | 0.41 | 0.88 |
| Gender |  |  | 2.84 | 2 | 0.24 |  |
| Female | -0.02 | 0.79 | 0.01 | 1 | 0.98 | 0.98 |
| Male | -1.16 | 0.97 | 1.43 | 1 | 0.23 | 0.31 |
| Model Summary | Log Likelihood | | Cox & Snell *R*^2^ | | Nagelkerke *R*^2^ | |
|  | 109.27 | | 0.20 | | 0.28 | |

**Table S8.** Logistic Regression Analysis Predicting Adolescent Endorsement of Text/Call Log for Clinical Practice

|  |  | | |  |  | |
| --- | --- | --- | --- | --- | --- | --- |
| Predictor | β | *SE* β | Wald’s χ^2^ | *df* | *p* | *e^β^* |
| Constant | 0.14 | 2.56 | 0.01 | 1 | 0.96 | 1.15 |
| Utility | 1.25 | 0.25 | 24.39 | 1 | <0.001 | 3.50 |
| Age | -0.18 | 0.16 | 1.33 | 1 | 0.25 | 0.84 |
| Gender |  |  | 0.04 | 2 | 0.98 |  |
| Female | -0.07 | 0.82 | 0.01 | 1 | 0.93 | 0.93 |
| Male | 0.19 | 1.04 | 0.04 | 1 | 0.85 | 0.82 |
| Model Summary | Log Likelihood | | Cox & Snell *R*^2^ | | Nagelkerke *R*^2^ | |
|  | 103.14 | | 0.30 | | 0.40 | |

**Table S9.** Logistic Regression Analysis Predicting Adolescent Endorsement of Text/Call Log for Research Purposes

|  |  | | |  |  | |
| --- | --- | --- | --- | --- | --- | --- |
| Predictor | β | *SE* β | Wald’s χ^2^ | *df* | *p* | *e^β^* |
| Constant | 0.15 | 2.56 | 0.00 | 1 | 0.95 | 1.16 |
| Utility | 1.24 | 0.26 | 23.68 | 1 | <0.001 | 3.46 |
| Age | -0.22 | 0.16 | 1.88 | 1 | 0.17 | 0.81 |
| Gender |  |  | 0.97 | 2 | 0.62 |  |
| Female | 0.74 | 0.81 | 0.83 | 1 | 0.36 | 2.09 |
| Male | 0.37 | 1.02 | 0.13 | 1 | 0.72 | 1.45 |
| Model Summary | Log Likelihood | | Cox & Snell *R*^2^ | | Nagelkerke *R*^2^ | |
|  | 102.42 | | 0.30 | | 0.40 | |

**Table S10.** Logistic Regression Analysis Predicting Adolescent Endorsement of Keyboard for Clinical Practice

|  |  | | |  |  | |
| --- | --- | --- | --- | --- | --- | --- |
| Predictor | β | *SE* β | Wald’s χ^2^ | *df* | *p* | *e^β^* |
| Constant | -6.73 | 2.79 | 5.84 | 1 | 0.02 | 0.01 |
| Utility | 0.98 | 0.23 | 18.08 | 1 | <0.001 | 2.67 |
| Age | 0.23 | 0.15 | 2.28 | 1 | 0.13 | 1.26 |
| Gender |  |  | 0.15 | 2 | 0.93 |  |
| Female | 0.08 | 0.75 | 0.01 | 1 | 0.92 | 1.08 |
| Male | 0.32 | 0.95 | 0.11 | 1 | 0.74 | 1.38 |
| Model Summary | Log Likelihood | | Cox & Snell *R*^2^ | | Nagelkerke *R*^2^ | |
|  | 108.95 | | 0.21 | | 0.28 | |

**Table S11.** Logistic Regression Analysis Predicting Adolescent Endorsement of Keyboard for Research Purposes

|  |  | | |  |  | |
| --- | --- | --- | --- | --- | --- | --- |
| Predictor | β | *SE* β | Wald’s χ^2^ | *df* | *p* | *e^β^* |
| Constant | -2.05 | 2.42 | 0.72 | 1 | 0.40 | 0.13 |
| Utility | 0.78 | 0.21 | 14.08 | 1 | <0.001 | 2.18 |
| Age | 0.01 | 0.14 | 0.00 | 1 | 0.99 | 1.00 |
| Gender |  |  | 0.10 | 2 | 0.95 |  |
| Female | -0.09 | 0.72 | 0.01 | 1 | 0.91 | 0.92 |
| Male | -0.26 | 0.92 | 0.08 | 1 | 0.77 | 0.77 |
| Model Summary | Log Likelihood | | Cox & Snell *R*^2^ | | Nagelkerke *R*^2^ | |
|  | 121.99 | | 0.15 | | 0.21 | |

**Table S12.** Logistic Regression Analysis Predicting Adolescent Endorsement of Microphone for Clinical Practice

|  |  | | |  |  | |
| --- | --- | --- | --- | --- | --- | --- |
| Predictor | β | *SE* β | Wald’s χ^2^ | *df* | *p* | *e^β^* |
| Constant | -3.39 | 2.97 | 1.31 | 1 | 0.25 | 0.03 |
| Utility | 1.32 | 0.27 | 24.40 | 1 | <0.001 | 3.73 |
| Age | -0.07 | 0.15 | 0.19 | 1 | 0.66 | 0.93 |
| Gender |  |  | 0.57 | 2 | 0.75 |  |
| Female | 0.49 | 0.94 | 0.27 | 1 | 0.61 | 1.63 |
| Male | 0.86 | 1.14 | 0.56 | 1 | 0.45 | 2.35 |
| Model Summary | Log Likelihood | | Cox & Snell *R*^2^ | | Nagelkerke *R*^2^ | |
|  | 93.60 | | 0.32 | | 0.44 | |

**Table S13.** Logistic Regression Analysis Predicting Adolescent Endorsement of Microphone for Research Purposes

|  |  | | |  |  | |
| --- | --- | --- | --- | --- | --- | --- |
| Predictor | β | *SE* β | Wald’s χ^2^ | *df* | *p* | *e^β^* |
| Constant | -2.17 | 2.90 | 0.56 | 1 | 0.45 | 0.11 |
| Utility | 1.33 | 0.27 | 24.82 | 1 | <0.001 | 3.80 |
| Age | -0.15 | 0.17 | 0.83 | 1 | 0.36 | 0.86 |
| Gender |  |  | 1.43 | 2 | 0.49 |  |
| Female | 0.63 | 0.95 | 0.45 | 1 | 0.50 | 1.88 |
| Male | 1.31 | 1.14 | 1.33 | 1 | 0.35 | 3.72 |
| Model Summary | Log Likelihood | | Cox & Snell *R*^2^ | | Nagelkerke *R*^2^ | |
|  | 94.74 | | 0.33 | | 0.45 | |

**Table S14.** Logistic Regression Analysis Predicting Adolescent Endorsement of Light for Clinical Practice

|  |  | | |  |  | |
| --- | --- | --- | --- | --- | --- | --- |
| Predictor | β | *SE* β | Wald’s χ^2^ | *df* | *p* | *e^β^* |
| Constant | -2.64 | 2.80 | 0.89 | 1 | 0.35 | 0.07 |
| Utility | 1.01 | 0.25 | 17.90 | 1 | <0.001 | 2.90 |
| Age | 0.02 | 0.16 | 0.02 | 1 | 0.89 | 1.02 |
| Gender |  |  | 3.12 | 2 | 0.21 |  |
| Female | 0.73 | 0.94 | 0.62 | 1 | 0.43 | 2.08 |
| Male | -0.54 | 1.09 | 0.24 | 1 | 0.62 | 0.59 |
| Model Summary | Log Likelihood | | Cox & Snell *R*^2^ | | Nagelkerke *R*^2^ | |
|  | 91.21 | | 0.23 | | 0.34 | |

**Table S15.** Logistic Regression Analysis Predicting Adolescent Endorsement of Light for Research Purposes

|  |  | | |  |  | |
| --- | --- | --- | --- | --- | --- | --- |
| Predictor | β | *SE* β | Wald’s χ^2^ | *df* | *p* | *e^β^* |
| Constant | -1.16 | 2.80 | 0.17 | 1 | 0.68 | 0.31 |
| Utility | 1.15 | 0.26 | 19.67 | 1 | <0.001 | 3.15 |
| Age | -0.09 | 0.16 | 0.30 | 1 | 0.58 | 0.91 |
| Gender |  |  | 2.92 | 2 | 0.23 |  |
| Female | 0.72 | 0.94 | 0.58 | 1 | 0.45 | 2.05 |
| Male | -0.52 | 1.10 | 0.23 | 1 | 0.63 | 0.59 |
| Model Summary | Log Likelihood | | Cox & Snell *R*^2^ | | Nagelkerke *R*^2^ | |
|  | 89.72 | | 0.26 | | 0.37 | |

**Table S16.** Logistic Regression Analysis Predicting Adolescent Endorsement of Screen for Clinical Practice

|  |  | | |  |  | |
| --- | --- | --- | --- | --- | --- | --- |
| Predictor | β | *SE* β | Wald’s χ^2^ | *df* | *p* | *e^β^* |
| Constant | -2.42 | 2.76 | 0.77 | 1 | 0.38 | 0.09 |
| Utility | 1.30 | 0.29 | 20.28 | 1 | <0.001 | 3.66 |
| Age | 0.01 | 0.16 | 0.00 | 1 | 0.99 | 1.01 |
| Gender |  |  | 0.02 | 2 | 0.99 |  |
| Female | 0.07 | 0.91 | 0.01 | 1 | 0.94 | 1.07 |
| Male | 0.15 | 1.14 | 0.02 | 1 | 0.90 | 1.16 |
| Model Summary | Log Likelihood | | Cox & Snell *R*^2^ | | Nagelkerke *R*^2^ | |
|  | 88.59 | | 0.26 | | 0.38 | |

**Table S17.** Logistic Regression Analysis Predicting Adolescent Endorsement of Screen for Research Purposes

|  |  | | |  |  | |
| --- | --- | --- | --- | --- | --- | --- |
| Predictor | β | *SE* β | Wald’s χ^2^ | *df* | *p* | *e^β^* |
| Constant | -0.66 | 2.66 | 0.06 | 1 | 0.80 | 0.52 |
| Utility | 1.22 | 0.27 | 20.33 | 1 | <0.001 | 3.39 |
| Age | -0.10 | 0.16 | 0.36 | 1 | 0.55 | 0.91 |
| Gender |  |  | 0.13 | 2 | 0.94 |  |
| Female | -0.19 | 0.89 | 0.04 | 1 | 0.83 | 0.83 |
| Male | 0.06 | 1.12 | 0.01 | 1 | 0.96 | 1.06 |
| Model Summary | Log Likelihood | | Cox & Snell *R*^2^ | | Nagelkerke *R*^2^ | |
|  | 93.94 | | 0.25 | | 0.36 | |

**Table S18.** Logistic Regression Analysis Predicting Adolescent Endorsement of GPS for Clinical Practice

|  |  | | |  |  | |
| --- | --- | --- | --- | --- | --- | --- |
| Predictor | β | *SE* β | Wald’s χ^2^ | *df* | *p* | *e^β^* |
| Constant | 0.05 | 2.64 | 0.00 | 1 | 0.98 | 1.05 |
| Utility | 1.24 | 0.25 | 24.44 | 1 | <0.001 | 3.46 |
| Age | -0.23 | 0.16 | 2.10 | 1 | 0.15 | 0.79 |
| Gender |  |  | 0.72 | 2 | 0.70 |  |
| Female | 0.45 | 0.83 | 0.32 | 1 | 0.57 | 1.60 |
| Male | -0.03 | 1.02 | 0.00 | 1 | 0.98 | 0.97 |
| Model Summary | Log Likelihood | | Cox & Snell *R*^2^ | | Nagelkerke *R*^2^ | |
|  | 99.30 | | 0.32 | | 0.43 | |

**Table S19.** Logistic Regression Analysis Predicting Adolescent Endorsement of GPS for Research Purposes

|  |  | | |  |  | |
| --- | --- | --- | --- | --- | --- | --- |
| Predictor | β | *SE* β | Wald’s χ^2^ | *df* | *p* | *e^β^* |
| Constant | 1.17 | 2.67 | 0.19 | 1 | 0.66 | 3.23 |
| Utility | 1.13 | 0.24 | 22.18 | 1 | <0.001 | 3.08 |
| Age | -0.21 | 0.16 | 1.69 | 1 | 0.19 | 0.81 |
| Gender |  |  | 1.50 | 2 | 0.47 |  |
| Female | -0.59 | 0.86 | 0.47 | 1 | 0.50 | 0.56 |
| Male | -1.23 | 1.04 | 1.41 | 1 | 0.24 | 0.29 |
| Model Summary | Log Likelihood | | Cox & Snell *R*^2^ | | Nagelkerke *R*^2^ | |
|  | 101.36 | | 0.29 | | 0.39 | |

**Table S20.** Logistic Regression Analysis Predicting Parent Endorsement of Accelerometer for Clinical Practice

|  |  | | |  |  | |
| --- | --- | --- | --- | --- | --- | --- |
| Predictor | β | *SE* β | Wald’s χ^2^ | *df* | *p* | *e^β^* |
| Constant | 18.10 | 1164.76 | 0.00 | 1 | 0.99 | 727158.20 |
| Utility | 1.22 | 0.33 | 13.66 | 1 | <0.001 | 3.39 |
| Age | -0.05 | 0.06 | 0.74 | 1 | 0.39 | 0.95 |
| Education | 0.48 | 0.22 | 4.69 | 1 | 0.03 | 1.62 |
| Gender (Female) | -19.99 | 1164.76 | 0.00 | 1 | 0.99 | 0.00 |
| Model Summary | Log Likelihood | | Cox & Snell *R*^2^ | | Nagelkerke *R*^2^ | |
|  | 64.73 | | 0.25 | | 0.41 | |

**Table S21.** Logistic Regression Analysis Predicting Parent Endorsement of Accelerometer for Research Purposes

|  |  | | |  |  | |
| --- | --- | --- | --- | --- | --- | --- |
| Predictor | β | *SE* β | Wald’s χ^2^ | *df* | *p* | *e^β^* |
| Constant | 18.10 | 1164.76 | 0.00 | 1 | 0.99 | 727158.20 |
| Utility | 1.22 | 0.33 | 13.66 | 1 | <0.001 | 3.93 |
| Age | -0.05 | 0.06 | 0.74 | 1 | 0.39 | 0.95 |
| Education | 0.48 | 0.22 | 4.69 | 1 | 0.03 | 1.62 |
| Gender (Female) | -19.98 | 1164.76 | 0.00 | 1 | 0.99 | 0.00 |
| Model Summary | Log Likelihood | | Cox & Snell *R*^2^ | | Nagelkerke *R*^2^ | |
|  | 64.73 | | 0.25 | | 0.41 | |

**Table S22.** Logistic Regression Analysis Predicting Parent Endorsement of Applications for Clinical Practice

|  |  | | |  |  | |
| --- | --- | --- | --- | --- | --- | --- |
| Predictor | β | *SE* β | Wald’s χ^2^ | *df* | *p* | *e^β^* |
| Constant | 16.64 | 1134.98 | 0.00 | 1 | 0.99 | 169036.30 |
| Utility | 1.52 | 0.39 | 15.31 | 1 | <0.001 | 4.59 |
| Age | -0.05 | 0.07 | 0.50 | 1 | 0.48 | 0.95 |
| Education | 0.58 | 0.25 | 5.59 | 1 | 0.02 | 1.79 |
| Gender (Female) | -19.96 | 1134.98 | 0.00 | 1 | 0.99 | 0.00 |
| Model Summary | Log Likelihood | | Cox & Snell *R*^2^ | | Nagelkerke *R*^2^ | |
|  | 55.22 | | 0.30 | | 0.50 | |

**Table S23.** Logistic Regression Analysis Predicting Parent Endorsement of Applications for Research Purposes

|  |  | | |  |  | |
| --- | --- | --- | --- | --- | --- | --- |
| Predictor | β | *SE* β | Wald’s χ^2^ | *df* | *p* | *e^β^* |
| Constant | 16.80 | 1169.90 | 0.00 | 1 | 0.99 | 197099.30 |
| Utility | 1.93 | 0.44 | 19.16 | 1 | <0.001 | 6.91 |
| Age | -0.02 | 0.06 | 0.15 | 1 | 0.70 | 0.98 |
| Education | 0.31 | 0.21 | 2.18 | 1 | 0.14 | 1.37 |
| Gender (Female) | -20.54 | 1169.90 | 0.00 | 1 | 0.99 | 0.00 |
| Model Summary | Log Likelihood | | Cox & Snell *R*^2^ | | Nagelkerke *R*^2^ | |
|  | 66.75 | | 0.43 | | 0.61 | |

**Table S24.** Logistic Regression Analysis Predicting Parent Endorsement of Bluetooth for Clinical Practice

|  |  | | |  |  | |
| --- | --- | --- | --- | --- | --- | --- |
| Predictor | β | *SE* β | Wald’s χ^2^ | *df* | *p* | *e^β^* |
| Constant | -3.03 | 2.40 | 1.60 | 1 | 0.21 | 0.05 |
| Utility | 1.00 | 0.26 | 15.11 | 1 | <0.001 | 2.73 |
| Age | 0.03 | 0.04 | 0.42 | 1 | 0.52 | 1.03 |
| Education | 0.26 | 0.19 | 2.02 | 1 | 0.16 | 1.30 |
| Gender (Female) | -1.17 | 1.17 | 1.01 | 1 | 0.32 | 0.31 |
| Model Summary | Log Likelihood | | Cox & Snell *R*^2^ | | Nagelkerke *R*^2^ | |
|  | 100.09 | | 0.26 | | 0.35 | |

**Table S25.** Logistic Regression Analysis Predicting Parent Endorsement of Bluetooth for Research Purposes

|  |  | | |  |  | |
| --- | --- | --- | --- | --- | --- | --- |
| Predictor | β | *SE* β | Wald’s χ^2^ | *df* | *p* | *e^β^* |
| Constant | -2.68 | 2.40 | 1.25 | 1 | 0.26 | 0.07 |
| Utility | 0.82 | 0.24 | 11.99 | 1 | <0.001 | 2.27 |
| Age | 0.01 | 0.04 | 0.12 | 1 | 0.73 | 1.02 |
| Education | 0.37 | 0.18 | 4.01 | 1 | 0.05 | 1.44 |
| Gender (Female) | -1.02 | 1.14 | 0.80 | 1 | 0.37 | 0.36 |
| Model Summary | Log Likelihood | | Cox & Snell *R*^2^ | | Nagelkerke *R*^2^ | |
|  | 103.77 | | 0.23 | | 0.31 | |

**Table S26.** Logistic Regression Analysis Predicting Parent Endorsement of Text/Call Log for Clinical Practice

|  |  | | |  |  | |
| --- | --- | --- | --- | --- | --- | --- |
| Predictor | β | *SE* β | Wald’s χ^2^ | *df* | *p* | *e^β^* |
| Constant | -9.07 | 2.86 | 10.05 | 1 | 0.01 | 0.00 |
| Utility | 1.86 | 0.39 | 22.57 | 1 | <0.001 | 6.41 |
| Age | 0.11 | 0.05 | 4.62 | 1 | 0.03 | 1.11 |
| Education | 0.04 | 0.23 | 0.03 | 1 | 0.85 | 1.04 |
| Gender (Female) | 0.23 | 1.21 | 0.04 | 1 | 0.85 | 1.26 |
| Model Summary | Log Likelihood | | Cox & Snell *R*^2^ | | Nagelkerke *R*^2^ | |
|  | 72.79 | | 0.41 | | 0.57 | |

**Table S27.** Logistic Regression Analysis Predicting Parent Endorsement of Text/Call Log for Research Purposes

|  |  | | |  |  | |
| --- | --- | --- | --- | --- | --- | --- |
| Predictor | β | *SE* β | Wald’s χ^2^ | *df* | *p* | *e^β^* |
| Constant | -6.52 | 2.53 | 6.66 | 1 | 0.01 | 0.00 |
| Utility | 1.44 | 0.31 | 21.87 | 1 | <0.001 | 4.22 |
| Age | 0.06 | 0.04 | 1.97 | 1 | 0.16 | 1.06 |
| Education | 0.11 | 0.21 | 0.28 | 1 | 0.60 | 1.11 |
| Gender (Female) | 0.29 | 1.08 | 0.07 | 1 | 0.79 | 1.34 |
| Model Summary | Log Likelihood | | Cox & Snell *R*^2^ | | Nagelkerke *R*^2^ | |
|  | 85.13 | | 0.35 | | 0.48 | |

**Table S28.** Logistic Regression Analysis Predicting Parent Endorsement of Keyboard for Clinical Practice

|  |  | | |  |  | |
| --- | --- | --- | --- | --- | --- | --- |
| Predictor | β | *SE* β | Wald’s χ^2^ | *df* | *p* | *e^β^* |
| Constant | -6.56 | 2.46 | 7.13 | 1 | 0.01 | 0.00 |
| Utility | 1.42 | 0.30 | 22.94 | 1 | <0.001 | 4.15 |
| Age | 0.06 | 0.04 | 2.04 | 1 | 0.15 | 1.06 |
| Education | 0.03 | 0.20 | 0.02 | 1 | 0.89 | 1.03 |
| Gender (Female) | 0.09 | 0.88 | 0.01 | 1 | 0.92 | 1.09 |
| Model Summary | Log Likelihood | | Cox & Snell *R*^2^ | | Nagelkerke *R*^2^ | |
|  | 94.69 | | 0.34 | | 0.45 | |

**Table S29.** Logistic Regression Analysis Predicting Parent Endorsement of Keyboard for Research Purposes

|  |  | | |  |  | |
| --- | --- | --- | --- | --- | --- | --- |
| Predictor | β | *SE* β | Wald’s χ^2^ | *df* | *p* | *e^β^* |
| Constant | -5.37 | 2.84 | 3.59 | 1 | 0.06 | 0.01 |
| Utility | 1.75 | 0.35 | 24.68 | 1 | <0.001 | 5.75 |
| Age | 0.03 | 0.05 | 0.39 | 1 | 0.53 | 1.03 |
| Education | -0.01 | 0.23 | 0.00 | 1 | 0.99 | 0.99 |
| Gender (Female) | -0.30 | 1.00 | 0.09 | 1 | 0.77 | 0.74 |
| Model Summary | Log Likelihood | | Cox & Snell *R*^2^ | | Nagelkerke *R*^2^ | |
|  | 82.45 | | 0.40 | | 0.54 | |

**Table S30.** Logistic Regression Analysis Predicting Parent Endorsement of Microphone for Clinical Practice

|  |  | | |  |  | |
| --- | --- | --- | --- | --- | --- | --- |
| Predictor | β | *SE* β | Wald’s χ^2^ | *df* | *p* | *e^β^* |
| Constant | -1.34 | 2.62 | 0.26 | 1 | 0.61 | 0.26 |
| Utility | 0.99 | 0.23 | 18.73 | 1 | <0.001 | 2.68 |
| Age | -0.01 | 0.05 | 0.01 | 1 | 0.93 | 0.99 |
| Education | 0.07 | 0.19 | 0.15 | 1 | 0.70 | 1.08 |
| Gender (Female) | -1.52 | 1.17 | 1.68 | 1 | 0.20 | 0.22 |
| Model Summary | Log Likelihood | | Cox & Snell *R*^2^ | | Nagelkerke *R*^2^ | |
|  | 101.58 | | 0.29 | | 0.39 | |

**Table S31.** Logistic Regression Analysis Predicting Parent Endorsement of Microphone for Research Purposes

|  |  | | |  |  | |
| --- | --- | --- | --- | --- | --- | --- |
| Predictor | β | *SE* β | Wald’s χ^2^ | *df* | *p* | *e^β^* |
| Constant | 0.25 | 2.69 | 0.01 | 1 | 0.93 | 1.28 |
| Utility | 0.90 | 0.22 | 17.35 | 1 | <0.001 | 2.47 |
| Age | -0.03 | 0.05 | 0.56 | 1 | 0.45 | 0.97 |
| Education | -1.62 | 1.19 | 1.86 | 1 | 0.17 | 0.20 |
| Gender (Female) | -1.62 | 1.19 | 1.86 | 1 | 0.17 | 0.20 |
| Model Summary | Log Likelihood | | Cox & Snell *R*^2^ | | Nagelkerke *R*^2^ | |
|  | 103.39 | | 0.28 | | 0.37 | |

**Table S32.** Logistic Regression Analysis Predicting Parent Endorsement of Light for Clinical Practice

|  |  | | |  |  | |
| --- | --- | --- | --- | --- | --- | --- |
| Predictor | β | *SE* β | Wald’s χ^2^ | *df* | *p* | *e^β^* |
| Constant | 16.17 | 1249.58 | 0.00 | 1 | 0.99 | 10566.60 |
| Utility | 1.08 | 0.29 | 14.08 | 1 | <0.001 | 2.96 |
| Age | -0.03 | 0.06 | 0.18 | 1 | 0.67 | 0.98 |
| Education | 0.55 | 0.23 | 5.75 | 1 | 0.02 | 1.73 |
| Gender (Female) | 19.09 | 1249.58 | 0.00 | 1 | 0.99 | 0.00 |
| Model Summary | Log Likelihood | | Cox & Snell *R*^2^ | | Nagelkerke *R*^2^ | |
|  | 68.33 | | 0.27 | | 0.43 | |

**Table S33.** Logistic Regression Analysis Predicting Parent Endorsement of Light for Research Purposes

|  |  | | |  |  | |
| --- | --- | --- | --- | --- | --- | --- |
| Predictor | β | *SE* β | Wald’s χ^2^ | *df* | *p* | *e^β^* |
| Constant | 20.96 | 1156.72 | 0.00 | 1 | 0.99 | 96897.99 |
| Utility | 0.93 | 0.26 | 12.24 | 1 | <0.001 | 2.52 |
| Age | -0.10 | 0.06 | 2.60 | 1 | 0.11 | 0.91 |
| Education | 0.54 | 0.22 | 6.11 | 1 | 0.01 | 1.71 |
| Gender (Female) | -19.62 | 1156.72 | 0.00 | 1 | 0.99 | 0.00 |
| Model Summary | Log Likelihood | | Cox & Snell *R*^2^ | | Nagelkerke *R*^2^ | |
|  | 70.67 | | 0.25 | | 0.40 | |

**Table S34.** Logistic Regression Analysis Predicting Parent Endorsement of Screen for Clinical Practice

|  |  | | |  |  | |
| --- | --- | --- | --- | --- | --- | --- |
| Predictor | β | *SE* β | Wald’s χ^2^ | *df* | *p* | *e^β^* |
| Constant | 19.11 | 1191.54 | 0.00 | 1 | 0.99 | 19976.99 |
| Utility | 1.01 | 0.28 | 13.25 | 1 | <0.001 | 2.73 |
| Age | -0.05 | 0.06 | 0.69 | 1 | 0.41 | 0.95 |
| Education | 0.40 | 0.21 | 3.59 | 1 | 0.06 | 1.50 |
| Gender (Female) | -19.63 | 1191.54 | 0.00 | 1 | 0.99 | 0.00 |
| Model Summary | Log Likelihood | | Cox & Snell *R*^2^ | | Nagelkerke *R*^2^ | |
|  | 64.78 | | 0.25 | | 0.41 | |

**Table S35.** Logistic Regression Analysis Predicting Parent Endorsement of Screen for Research Purposes

|  |  | | |  |  | |
| --- | --- | --- | --- | --- | --- | --- |
| Predictor | β | *SE* β | Wald’s χ^2^ | *df* | *p* | *e^β^* |
| Constant | 1.81 | 2.95 | 0.38 | 1 | 0.54 | 6.12 |
| Utility | 0.63 | 0.22 | 8.07 | 1 | 0.01 | 1.88 |
| Age | -0.08 | 0.05 | 2.37 | 1 | 0.12 | 0.92 |
| Education | 0.49 | 0.20 | 6.28 | 1 | 0.01 | 1.64 |
| Gender (Female) | -0.51 | 1.22 | 0.18 | 1 | 0.68 | 0.60 |
| Model Summary | Log Likelihood | | Cox & Snell *R*^2^ | | Nagelkerke *R*^2^ | |
|  | 81.39 | | 0.19 | | 0.29 | |

**Table S36.** Logistic Regression Analysis Predicting Parent Endorsement of GPS for Clinical Practice

|  |  | | |  |  | |
| --- | --- | --- | --- | --- | --- | --- |
| Predictor | β | *SE* β | Wald’s χ^2^ | *df* | *p* | *e^β^* |
| Constant | -4.02 | 3.05 | 1.73 | 1 | 0.19 | 0.02 |
| Utility | 1.29 | 0.25 | 26.21 | 1 | <0.001 | 3.64 |
| Age | 0.02 | 0.05 | 0.10 | 1 | 0.75 | 1.02 |
| Education | 0.26 | 0.22 | 1.41 | 1 | 0.24 | 1.30 |
| Gender (Female) | -1.04 | 1.30 | 0.64 | 1 | 0.42 | 0.35 |
| Model Summary | Log Likelihood | | Cox & Snell *R*^2^ | | Nagelkerke *R*^2^ | |
|  | 79.66 | | 0.41 | | 0.55 | |

**Table S37.** Logistic Regression Analysis Predicting Parent Endorsement of GPS for Research Purposes

|  |  | | |  |  | |
| --- | --- | --- | --- | --- | --- | --- |
| Predictor | β | *SE* β | Wald’s χ^2^ | *df* | *p* | *e^β^* |
| Constant | -0.74 | 3.07 | 0.06 | 1 | 0.81 | 0.48 |
| Utility | 1.20 | 0.24 | 25.03 | 1 | <0.001 | 3.30 |
| Age | -0.02 | 0.05 | 0.18 | 1 | 0.67 | 0.99 |
| Education | 0.09 | 0.21 | 0.18 | 1 | 0.67 | 1.09 |
| Gender (Female) | -1.51 | 1.39 | 1.18 | 1 | 0.28 | 0.22 |
| Model Summary | Log Likelihood | | Cox & Snell *R*^2^ | | Nagelkerke *R*^2^ | |
|  | 85.32 | | 0.38 | | 0.51 | |

**VII. COREQ Checklist**

**Consolidated criteria for reporting qualitative studies (COREQ): 32-item checklist**

| **No. Item** | **Guide questions/description** | **Reported on Page #** |
| --- | --- | --- |
| **Domain 1: Research team and reﬂexivity** |  |  |
| *Personal Characteristics* |  |  |
| 1. Interviewer/facilitator | Which author/s conducted the interview or focus group? | NA – this was a REDCap survey delivered virtually. |
| 2. Credentials | What were the researcher’s credentials? E.g. PhD, MD | NA – see above. |
| 3. Occupation | What was their occupation at the time of the study? | NA – see above. |
| 4. Gender | Was the researcher male or female? | NA – see above. |
| 5. Experience and training | What experience or training did the researcher have? | NA – see above. |
| *Relationship with participants* |  |  |
| 6. Relationship established | Was a relationship established prior to study commencement? | p.6 |
| 7. Participant knowledge of the interviewer | What did the participants know about the researcher? e.g. personal goals, reasons for doing the research | p.6 |
| 8. Interviewer characteristics | What characteristics were reported about the interviewer/facilitator? e.g. Bias, assumptions, reasons and interests in the research topic | NA – this was a REDCap survey delivered virtually. |

| **Domain 2: study design** |  |  |
| --- | --- | --- |
| *Theoretical framework* |  |  |
| 9. Methodological orientation and Theory | What methodological orientation was stated to underpin the study? e.g. grounded theory, discourse analysis, ethnography, phenomenology, content analysis | p.9 |
| *Participant selection* |  |  |
| 10. Sampling | How were participants selected? e.g. purposive, convenience, consecutive, snowball | p.7 |
| 11. Method of approach | How were participants approached? e.g. face-to-face, telephone, mail, email | p.6 |
| 12. Sample size | How many participants were in the study? | p.7 |
| 13. Non-participation | How many people refused to participate or dropped out? Reasons? | p.7 |
| *Setting* |  |  |
| 14. Setting of data collection | Where was the data collected? e.g. home, clinic, workplace | NA – this was a REDCap survey delivered virtually. |
| 15. Presence of non-participants | Was anyone else present besides the participants and researchers? | NA – see above. |
| 16. Description of sample | What are the important characteristics of the sample? e.g. demographic data, date | p.11 |
| *Data collection* |  |  |
| 17. Interview guide | Were questions, prompts, guides provided by the authors? Was it pilot tested? | p.7-8 |
| 18. Repeat interviews | Were repeat interviews carried out? If yes, how many? | NA – this was a one-time online survey. |
| 19. Audio/visual recording | Did the research use audio or visual recording to collect the data? | NA |
| 20. Field notes | Were ﬁeld notes made during and/or after the interview or focus group? | NA |
| 21. Duration | What was the duration of the interviews or focus group? | p.9 |
| 22. Data saturation | Was data saturation discussed? | p.10 |
| 23. Transcripts returned | Were transcripts returned to participants for comment and/or correction? | NA |
| **Domain 3: analysis and ﬁndings** |  |  |
| *Data analysis* |  |  |
| 24. Number of data coders | How many data coders coded the data? | p.10 |
| 25. Description of the coding tree | Did authors provide a description of the coding tree? | p.10 |
| 26. Derivation of themes | Were themes identiﬁed in advance or derived from the data? | p.10 |
| 27. Software | What software, if applicable, was used to manage the data? | p.10 |
| 28. Participant checking | Did participants provide feedback on the ﬁndings? | NA |
| *Reporting* |  |  |
| 29. Quotations presented | Were participant quotations presented to illustrate the themes/ﬁndings? Was each quotation identiﬁed? e.g. participant number | p.21-22 |
| 30. Data and ﬁndings consistent | Was there consistency between the data presented and the ﬁndings? | p.23-24 |
| 31. Clarity of major themes | Were major themes clearly presented in the ﬁndings? | p.25-27 |
| 32. Clarity of minor themes | Is there a description of diverse cases or discussion of minor themes? | p.26-28 |

**VIII. REDCap Survey**

See attached PDF.
